# Gold Nanoparticles: Multifunctional Properties, Synthesis, and Future Prospects

**DOI:** 10.3390/nano14221805

**Published:** 2024-11-11

**Authors:** Hatice Duman, Emir Akdaşçi, Furkan Eker, Mikhael Bechelany, Sercan Karav

**Affiliations:** 1 Department of Molecular Biology and Genetics, Çanakkale Onsekiz Mart University, Çanakkale 17100, Türkiye; hatice.duman@comu.edu.tr (H.D.); emirakdasci@gmail.com (E.A.); furkan.eker@stu.comu.edu.tr (F.E.); 2 Institut Européen des Membranes (IEM), UMR 5635, University Montpellier, ENSCM, CNRS, F-34095 Montpellier, France; 3 Functional Materials Group, Gulf University for Science and Technology (GUST), Masjid Al Aqsa Street, Mubarak Al-Abdullah 32093, Kuwait

**Keywords:** gold nanoparticles, green synthesis, chemical and physical synthesis, optical property, delivery, surface functionalization, toxicity

## Abstract

Gold nanoparticles (NPs) are among the most commonly employed metal NPs in biological applications, with distinctive physicochemical features. Their extraordinary optical properties, stemming from strong localized surface plasmon resonance (LSPR), contribute to the development of novel approaches in the areas of bioimaging, biosensing, and cancer research, especially for photothermal and photodynamic therapy. The ease of functionalization with various ligands provides a novel approach to the precise delivery of these molecules to targeted areas. Gold NPs’ ability to transfer heat and electricity positions them as valuable materials for advancing thermal management and electronic systems. Moreover, their inherent characteristics, such as inertness, give rise to the synthesis of novel antibacterial and antioxidant agents as they provide a biocompatible and low-toxicity approach. Chemical and physical synthesis methods are utilized to produce gold NPs. The pursuit of more ecologically sustainable and economically viable large-scale technologies, such as environmentally benign biological processes referred to as green/biological synthesis, has garnered increasing interest among global researchers. Green synthesis methods are more favorable than other synthesis techniques as they minimize the necessity for hazardous chemicals in the reduction process due to their simplicity, cost-effectiveness, energy efficiency, and biocompatibility. This article discusses the importance of gold NPs, their optical, conductivity, antibacterial, antioxidant, and anticancer properties, synthesis methods, contemporary uses, and biosafety, emphasizing the need to understand toxicology principles and green commercialization strategies.

## 1. Introduction

In recent years, traditional biomedical techniques have been effectively supplanted by contemporary nanotechnology approaches, offering enhanced accuracy, sensitivity, efficiency, and rapid measurement. Many studies have been conducted on gold NPs for biological applications because they have many useful physical and chemical properties, such as being easy to make, biocompatible, non-toxic, having a high surface-to-volume ratio, and being able to change their size. The size and form of gold NPs affect their physical properties and color diversity, while bulk gold has different attributes than nanoscale particles [1].

The distinctive characteristics of gold NPs have driven researchers to develop them for applications in catalysis, biolabeling, nonlinear photonic systems, and drug delivery [2]. The optical characteristics of gold NPs, characterized by their strong LSPR, enable utilization in advancing bioimaging and biosensing studies, along with commonly employed spectroscopic techniques such as Surface-Enhanced Raman Scattering (SERS), which enables precise detection of analytes at extremely low levels, down to the femtomolar and picomolar scales [3]. It also extends to cancer therapy where researchers are using gold NPs’ photothermal characteristics to improve the overall efficiency of photothermal treatment, especially to facilitate localized heating processes [4]. Free-moving electrons on gold NPs’ outer membrane provide high electrical conductivity efficiency by facilitating a conductive pathway. This has recently led to the development of novel devices in the electronics field, such as conductive inks, where gold NPs are used as additives and coating and doping materials [5]. Further, gold NPs’ high thermal conductivity is exploited by researchers for the production of thermal management systems, including heat pipes for electronics cooling, where rapid and even distribution of heat is a crucial requirement [6].

One of the most recognized research areas focusing on the extensive employment of gold NPs includes drug delivery. The facile synthesis of gold NPs, combined with their synergistic effects with various ligands, such as drugs, nucleic acids, chemotherapeutic agents, proteins, glycans, antibacterials, and photosensitizers, paves the way for the creation of highly efficient delivery platforms [7]. From another aspect, where gold NPs are not integrated with other molecules, there is an increasing amount of research highlighting the novel and nature-friendly antimicrobials, along with antioxidant and anticancer agents, synthesized using gold NPs [8,9,10]. This is mostly due to the recently developed green synthesis approaches, which take advantage of the gold NPs’ inherent properties, including low toxicity, high stability, and biocompatibility.

In the synthesis of gold NPs, two primary approaches are employed: top-down and bottom-up. Top-down approaches include the extraction of bulk material to form self-assembled nanoscale objects, whereas bottom-up approaches require the assembly of tiny atoms or reducing ions to form crucial nanostructures. One safe, effective, and energy-efficient way to manufacture NPs is through biological/green synthesis. This method uses a variety of biological resources, including eukaryotes and prokaryotes, to generate NPs in vivo. The bioreduction of metallic ions to NPs and the stability of these particles are significantly influenced by metabolites, which include proteins, fatty acids, carbohydrates, enzymes, and phenolic substances [11]. Therefore, numerous biological systems, including plants, bacteria, yeasts, and fungi, are actively investigated to find new pathways for the synthesis of safe nanoproducts for the manufacturing of gold NPs [12]. One of the greatest options for the large-scale production of gold NPs with well-defined size and morphology is plant-based green synthesis technology because of its affordable cost [13]. However, chemical methods are frequently employed because of their simplicity; nevertheless, for reduction, harmful chemicals are required. While biological approaches use the same reducing and functionalizing agents, chemical procedures offer a wider range of functionalization options for creating NPs with different functionalizing agents. Chemical synthesis methods control the size of the particles, whereas biological processes yield big particles. While chemical synthesis needs a high temperature, room temperature synthesis is a potential biological technique. On the other hand, environmental advantages, high-purity particles free of hazardous chemicals, exact control over NP size and shape, and simple industrial uses such as laser ablation or sputtering are all provided by the physical production of gold NPs. Its disadvantages over chemical synthesis include higher energy consumption, more expensive equipment, and lower yields [12,14].

Gold NPs have garnered significant interest in the realm of biomedicine and its applications. The substantial potential for future applications presented by the rapid growth of gold NP technology can be attributed to their diversified characteristics and huge volume-specific surface areas in comparison to bulk gold. Due to these characteristics, gold NPs are now important for the creation of superior nanoelectronic chips as well as a range of biomedical and environmental applications [15,16]. They have also been employed in the food and beverage sectors [17]. Additionally, the Food and Drug Administration (FDA) approved gold NPs for a variety of biomedical uses, which led to an expansion of application areas, including as medication carriers, in cancer therapy, and other biological uses [18]. The extensive everyday use of gold NPs may elevate the likelihood of human exposure to these NPs. Numerous investigations into the toxicological effects of gold NPs from both academic and commercial sources have been disclosed [19]. Despite its potential in medicinal, environmental, and industrial applications, evidence regarding the acute and long-term health impacts of manufactured NPs remains scarce. These NPs can traverse the bloodstream without eliciting immunological rejection, yet their diminutive size and surface charges raise concerns. Gold NPs, produced in diverse forms, sizes, and charges, possess notable physicochemical features that raise new health concerns. Currently, there is insufficient knowledge regarding the health impacts of gold NPs, and no regulatory safety guidelines exist for their hazardous qualities [20].

**Figure 1:**
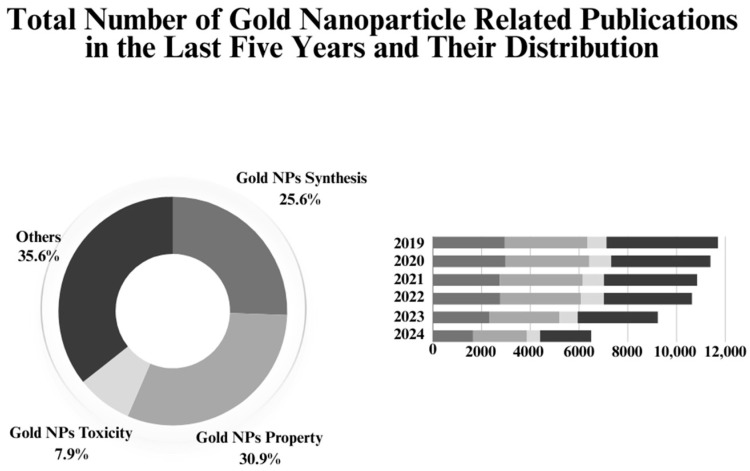
Comparison of the total number of gold-NP-related publications in the last five years [21].

Gold NPs are among the most frequently discussed nanomaterials in nanotechnology. According to the Web of Science (WoS) database, approximately 10,000 papers titled “gold nanoparticles” are published annually (Figure 1) [21]. Although this is a significant number, the total publications on gold NPs are relatively low in number compared to silver NPs, as discussed in our previous paper [22,23,24]. Silver NPs are widely researched in the current literature, including in combined and comparative studies involving gold NPs. One reason behind this research difference between these two NPs could be the challenges associated with green synthesis methods, particularly for gold NPs. Silver NPs have more established synthesis routes and demonstrate significant activity in certain areas. These challenges are well-documented in the literature, with a focus on missing points in optimizing the physicochemical properties of gold NPs during synthesis [25,26,27]. This may explain the pie chart showing the distribution of papers that include “property” and “synthesis” as keywords in their titles. It can be considered that the remaining articles (“others”) mainly focus on investigating gold NP applications, including biological activities. However, based on this, the current literature primarily explores synthesis methods and the physicochemical aspects of gold NPs, rather than their broad applications, as is common with silver NPs. Therefore, up-to-date reviews on gold NPs, especially those focusing on their synthesis processes and physical properties, are extremely crucial for the future of gold NP research.

This review paper presents an overview of the synthesis of gold NPs by the use of diverse synthesis methods, classified as physical, chemical, and biological/green synthesis approaches. It examines their varied properties. This paper examines the various parameters influencing the synthesis of gold NPs to achieve optimum sizes and shapes, as well as the characterization methods employed to gain a deeper understanding of their characteristics. The significance of gold NPs, their improved optical and conductivity qualities, and their antibacterial, antioxidant, and anticancer capabilities are all covered in this article. With an emphasis on gold NP research, this article examines the biosafety of gold NPs, underscoring potential hazardous effects on cells, tissues, and organs and stressing the necessity for a thorough understanding of and adherence to toxicology principles. It draws attention to the increasing need for environmentally friendly industrial applications and makes noteworthy recommendations for developments in green commercialization strategies.

## 2. Properties of Gold Nanoparticles

**Figure 2:**
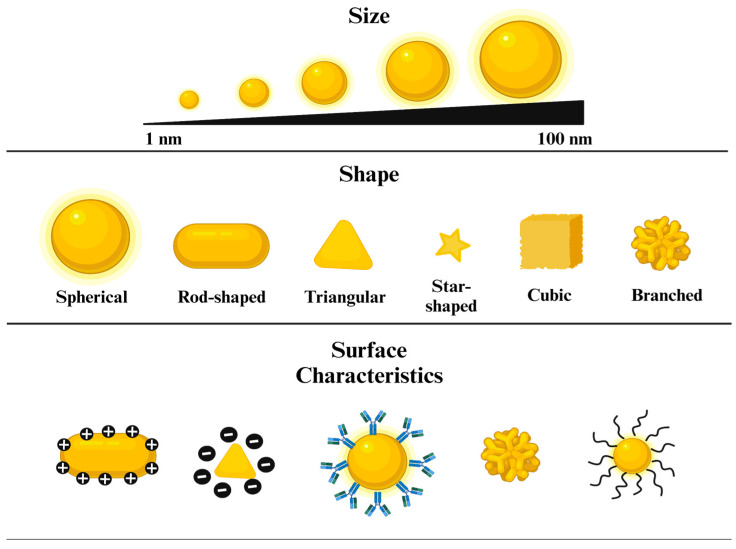
Gold NPs’ size ranges between 1 to 100 nm. They can have various shapes, including spherical, rod, triangular, star, cubic, or branched, depending on the synthesis method and further use. Their surface characteristics including surface charge can be manipulated using various approaches, such as pegylation and antibody modification to enhance the overall stability and biocompatibility of the NPs [30].

**Figure 3:**
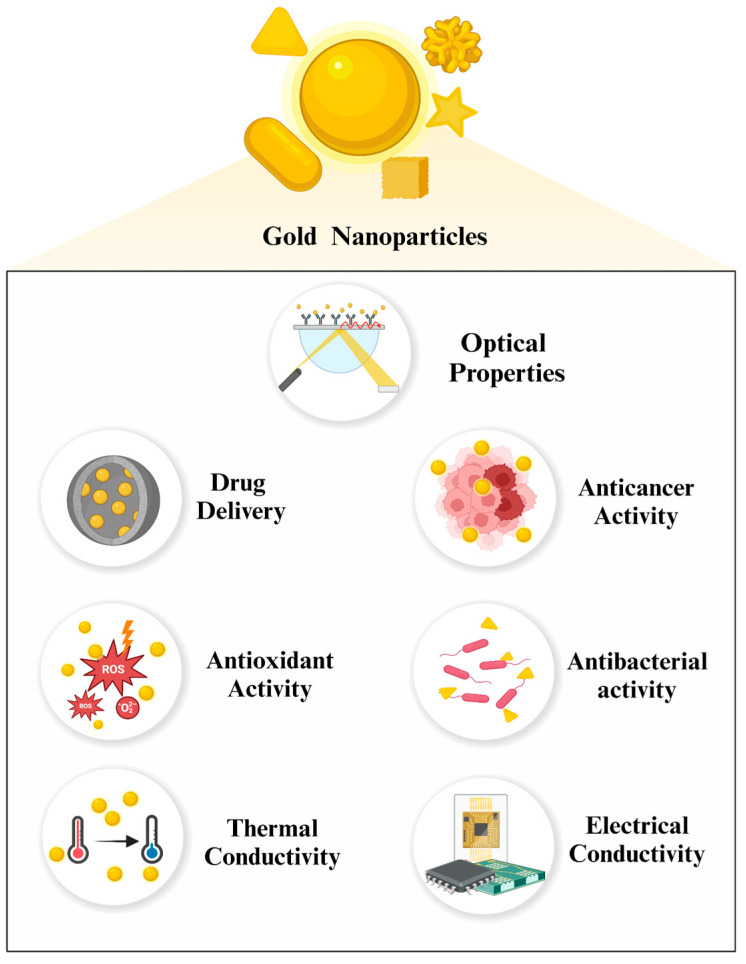
Highlighted properties of gold NPs [12].

Metal NPs possess remarkable physical and chemical attributes that differ significantly from their bulkier counterparts. In this manner, they have been widely utilized in diverse fields of science, especially in the biomedical area. The properties of gold NPs are heavily exploited in certain applications. Their superior optical properties, due to powerful LSPR, pave the way for utilization in bioimaging, biosensing, photothermal treatment (PTT), and photodynamic therapy (PDT). On the other hand, physical properties such as size, shape, and surface charge, combined with ease of functionalization with various biomolecules, facilitate the development of novel drug delivery systems [28]. In addition, their natural characteristics, including electrical and thermal conductivity, low toxicity, biocompatibility, and inertness, highlight gold NPs as preferable additives to cooling systems, anticancer and antioxidant agents, and anti-inflammatory nanoformulations [29,30]. Owing to their extraordinary characteristics, especially compared to other types of metal NPs, gold NPs were one of the most studied. In this section, we have pointed out the distinctive properties of gold NPs, from physical to biological (Figure 2), to underline their importance across multiple scientific disciplines (Figure 3).

### 2.1. Size

Gold NPs exhibit size-dependent physical, chemical, and biological characteristics that significantly determine their applicability in various fields [30]. It increases their interaction with their surroundings by providing a high surface-area-to-volume ratio, further enhancing their optical properties, catalytic activity, and biocompatibility [31]. For example, Shafiqa et al. investigated the dependence of surface plasmon resonance (SPR) concerning the size of gold nanospheres, which ranged from 20 to 100 nm. Results revealed that absorption efficiency increases with NP size, with a size of 70 nm demonstrating optimal efficiency compared to their counterparts [32]. Regarding catalytic behavior, Suchomel et al. explored the size-dependent activity of gold NPs, focusing on sizes varying from 6 to 22 nm. It has been shown that catalytic activity increased with decreasing size, which is primarily attributed to a higher surface-area-to-volume ratio [33].

Another aspect is that the size of the NPs is known to be a major determinant of their potential adverse effects. In this manner, Lee et al. investigated the size-dependent toxicity of gold NPs in human neural precursor cells and the rat cerebral cortex. Utilizing two groups of gold NPs, with sizes of 5 and 100 nm, they have demonstrated a correlation between size and toxic effects. Findings revealed that smaller NPs (5 nm) exhibited higher toxicity compared to larger ones (100 nm), evidenced by a significant reduction in cell viability [34].

### 2.2. Shape

The shape of the NPs plays a key role in the definition of their resultant properties and further uses [35]. Gold NPs can be fabricated in various shapes such as spheres, cubes, triangles, rods, and stars [36]. Each of these shapes comes with distinct advantages and disadvantages. To exemplify, Jiji et al. investigated the catalytic performance of differently shaped gold NPs for the reduction of p-nitroaniline. Findings demonstrated that gold nanorods (NRs) were superior in terms of catalytic efficiency compared to their spherical and dog bone counterparts [37]. From another perspective, Hameed et al. focused on the antibacterial activity of differently shaped gold NPs. Emphasizing the increased bactericidal efficiency at lower doses of NPs, they have shown that gold NPs interacted with foodborne bacterial pathogens, such as *Escherichia coli* (*E. coli)*, *Pseudomonas aeruginosa* (*P. aeruginosa*), and *Staphylococcus aureus* (*S. aureus*), in a shape-dependent manner. In more detail, gold nanocubes were most bactericidal in comparison to nanospheres and nanostars, indicated by a zero percent survival rate [38]. In addition, the shape of the gold NPs highly influences their optical traits. Hua et al. examined the nonlinear optical properties of gold NPs with varying shapes, including NRs, nanostars, and nanoshells. Utilizing Z-scan measurements and pump-probe spectroscopy, they have revealed that gold NRs exhibited the strongest saturable absorption while nanoshells showed the weakest response [39].

Moreover, shape is an important factor that affects the efficiency of gold NP-based delivery systems. One of the primary examples of this aspect includes the delivery of nucleic acids, such as small interfering RNAs (siRNAs). Accordingly, Morgan et al. demonstrated how various differently shaped gold NPs (nanoshells, nanocages, and NRs) influence the effectiveness of siRNA delivery. Their results highlighted that NRs were the most efficient in terms of the release of the attached siRNA strands, even though nanoshells could be loaded with three times more siRNA cargo [40]. In another study, Xie et al. evaluated the effect of NP shape concerning cellular uptake into RAW264.7 cells. Focusing on stars, rods, and triangles, they found that differently shaped gold NPs are prone to demonstrate cellular uptake through different pathways, with triangles being the most efficient among them [41]. These results not only highlighted the possible mechanisms of cellular uptake but also created a novel perspective for the further design of nanomaterials in drug delivery systems.

In addition, similar to their size, there are various studies in the current literature underlining the relationship between toxicity and the shape of the gold NPs. To name one, Sun et al. investigated the in vivo toxic effects and the biodistribution of sphere-, rod-, and cube-shaped gold NPs using a mouse model. Their findings showed that the ideal shape of the NPs was spherical since this showed the highest biocompatibility. On the other hand, rod-shaped NPs were observed as the most toxic, evidenced by increased adverse effects at lower concentrations of exposure [42]. In an in vitro study, Woźniak et al. examined the size- and shape-dependent cytotoxicity profiles of gold nanospheres, nanoflowers, NRs, nanostars, and nanoprisms on HEK293T and HeLa cell lines. It was observed that NRs and nanospheres were the most toxic since they led to the greatest decrease in the number of cells, specifically at the concentrations of 32, 100, and 300 µM. However, nanostars, nanoprisms, and nanoflowers remained less lethal at the same concentrations and only caused toxic effects at the highest doses after 72 h of exposure [43].

### 2.3. Surface Characteristics

#### 2.3.1. Surface Charge

Surface charge is particularly important in determining the stability, biodistribution, and interaction of NPs with their environment [44]. Typically, as a result of the negatively charged identity of the cell membrane, positively charged NPs interact more easily with cells compared to their negatively charged or neutral counterparts [45]. Taking this as an advantage, like most other NPs, positively charged gold NPs are employed as efficient delivery agents in the biomedical area, considering their increased cellular uptake characteristics [18]. As an example, Noh et al. employed cationic gold NPs for the delivery of plasmid DNA (pVAXmIL-2) into C2C12 cells. Through experiments, they examined gold NP–plasmid DNA complexes with various complexation rates and reported an enhancement in transfection efficiency, particularly at the ratio of 2400:1. Furthermore, a 4-fold increase in expression levels was also reported after intramuscular administration of the NP–DNA complex compared to DNA alone [46].

On the other hand, although anionic gold NPs have relatively low efficiency in terms of cellular uptake, they have also been widely investigated. In such a study, Lee et al. explored the influence of surface charge modifications of gold NRs on skin penetration. Using transmission electron microscopy (TEM), they have measured the ability of differently charged NPs to penetrate the skin. Results of the image analysis revealed that negatively charged gold NPs penetrated more rapidly than positive ones, emphasizing the importance of surface chemistry for the development of suitable approaches in the near future [47].

In addition, negatively charged gold NPs have proven to be less toxic. In this manner, Bozich et al. assessed the toxic effects of gold NPs on *Daphnia Magna* by focusing on different variables such as surface chemistry, charge, and the ligand type. Following both chronic and acute assays, results demonstrated that positively charged gold NPs were more toxic in comparison to their negatively charged counterparts [48]. Similarly, Feng et al. focused on the adverse effects of positively and negatively charged gold NPs on Gram-negative *Shewanella oneidensis* MR-1 and Gram-positive *Bacillus subtilis* (*B. subtilis*). Findings revealed that cationic gold NPs, especially PAH-gold NPs, were more toxic to both types of bacteria [49]. Overall, these results are primarily attributed to the preferential binding of positively charged NPs towards the negatively charged surfaces of cell membranes.

#### 2.3.2. Surface Functionalization

Manipulation of the surface characteristics of NPs enables their utilization in various areas since it provides a promising opportunity for novel uses [50]. In the case of gold NPs, various approaches such as covalent attachment (thiolation) [51], electrostatic interaction (citrate capping) [52], bioconjugation (with proteins, antibodies, and DNA) [53], and coating with polymers (PEG and chitosan) [54] can be followed. These interactions, as a result of the strong affinity of gold NPs towards functional groups, facilitate the targeted delivery of these NPs into cells, as well as biosensing and bioimaging applications [55].

In their research, Encabo-Berzosa et al. investigated the efficiency of PEG-functionalized polyethylenimine (PEI) gold NPs, as nonviral vectors, in comparison to commercially available lipoplexes. Bounding NPs to differently sized plasmids, from 4 to 40 kbp, their performance was evaluated on HeLa and Hek293t cells. Results indicated the potential applicability of gold NP complexes, as they have shown the same or increased levels of expression compared to commercially available alternatives. It was also mentioned that pegylation of the gold NPs not only reduced the toxic effects arising from the PEI but also increased the dispersion of the NPs in the culture media [56]. In another study, Medley et al. synthesized aptamer-conjugated gold NPs to address the limitations associated with the detection of cancerous cells, including high-priced instrumentation and time-consuming methods. Developing a novel colorimetric assay, they were able to differentiate between target and control cells by observing the color change arising from the assembly of gold NPs on the cell surface. This novel approach emphasized the potential use of functionalized gold NPs in oncology, along with adding a highly sensitive and cost-effective method to the current literature [57]. In addition, functionalization of the surface characteristics of gold NPs can be applied to mitigate their toxic effects, one of the major concerns associated with nanomaterials [58].

### 2.4. Optical Properties

In previous years, gold NPs have been the main focus of numerous research in the diagnostics area as a result of their extraordinary optical properties [59]. The most highlighted of these properties is LSPR, which is defined as the collective oscillating motion of conduction electrons near the NP surface when illuminated with light [60]. This phenomenon is primarily known for enabling gold NPs to absorb and scatter light in a broad range of the spectrum, consequently creating the basis for novel approaches in imaging, sensing, and labeling studies [61]. This distinctive property, along with their high biocompatibility and surface area, makes gold NPs preferable to other common metal NPs in biosensor development [62]. For example, functionalized gold NPs are being extensively used in bioimaging research, especially in oncology for the diagnosis of tumors, as they improve contrast and resolution in a biological environment [63]. In the same manner, there is a lot of research in the current literature focusing on the incorporation of gold NPs into sensing devices, with the aim of developing highly sensitive biosensors that demonstrate lower limits of detection [64].

The optical properties of gold NPs are highly influenced by their morphological characteristics, including their shape. To exemplify, differently shaped gold NPs have been shown to exhibit dissimilar LSPR peaks [65]. While nanospheres exhibit one unique LSPR band at around 520 nm, NRs possess two split bands, transverse LSPR (t-LSPR) and longitudinal LSPR (l-LSPR), owing to their two-dimensional structure. Although t-LSPR peaks are located at around 520 nm regardless of the dimension, l-LSPR peaks can be detected at higher wavelengths, from 650 to 1050 nm, depending on the NP aspect ratio [66]. On the other hand, branched or complex structures (including nanostars), with their asymmetric shapes, create multiple sharp resonance peaks that give rise to unique optical traits [67].

LSPR is also responsible for the distinctive colors of gold NPs. Since surface absorption bands are known to be size- and shape-dependent, a variation in these parameters causes a shift in the absorption peaks; thus, a change in color is observed [68]. Generally, gold NPs show visible colors, from red to purple, when dispersed in a liquid medium and demonstrate absorption peaks at 500 to 550 nm. However, when the particle size increases, the color of the solution differs depending on the size of the NPs [69]. In line with this, Zeng et al. utilized citrate-stabilized gold NPs for the visual detection of melamine, which is a non-protein nitrogen source illegally integrated into milk products to increase the measured concentration of total protein [70]. Benefiting from the strong electrostatic interaction between melamine and the gold NPs, melamine levels down to 10 ppb were successfully detected upon a color change over a short period of time. It was also emphasized that the size of the gold NPs plays an important role in colorimetric detection, evidenced by an improvement in sensitivity, from 15 to 5 ppm, when the size of the nanomaterials increased from 5 to 30 nm.

To summarize, researchers take advantage of the optical properties of gold NPs in various applications, primarily in rapid diagnostic tests, such as colorimetric studies and immunoassays [71].

Moreover, the LSPR characteristics of gold NPs extend to bioanalytical techniques such as SERS. SERS is a specialized form of Raman spectroscopy that is based on the principle of enhancing Raman scattering signals by using metal surfaces (or NPs) for the sensitive detection of molecular substances at lower concentrations, even down to 10^−12^ (picomolar) and 10^−15^ (femtomolar) levels [72]. The employment of gold NPs as SERS substrates has been widely studied thanks to their strong LSPR. As an example, Xu et al. developed a novel sensor for the detection and analysis of pesticides, 2,4-dichlorophenoxyacetic acid (2,4-D), pymetrozine, and thiamethoxam in food. Incorporating gold NPs within the mesoporous silica film in a densely packed form, researchers established a highly sensitive and stable SERS substrate containing multiple “hot spots”. These hot spots successfully amplified the Raman signals and allowed for the detection of trace amounts of pesticides, supported by a recorded limit of detection as low as 0.79 pg/mL for 2,4-D [73].

Additionally, gold NPs exhibit remarkable photothermal properties that allow for the development of novel approaches, especially in oncology [74]. One of these approaches includes PTT, where the energy of light absorbed by the NPs is converted into heat in order to trigger cellular apoptosis [75]. As an example, Faid et al. showed the effectiveness of citrate-capped gold NPs in PTT as photothermal agents. Conducting experiments on a breast cancer cell line (MCF-7), they revealed the higher photothermal efficiency of gold NPs in comparison to irradiation with a 532 nm laser alone, supported by a significant 34% reduction in cell viability [76].

Another therapeutic approach where gold NPs are utilized is PDT. PDT is based on the combined use of molecular oxygen and photosensitizers for the inhibition of cancer cell proliferation [77]. During the process, a photosensitizing agent is administered into the target tissue and illuminated with light. Upon exposure to specific wavelengths, the photosensitizing agent becomes activated and induces the generation of reactive oxygen species (ROS), eventually causing the death of cancerous cells [78]. In this context, gold NPs are commonly used to promote the efficiency of PDT. For example, Eshghi et al. synthesized Protoporphyrin IX (PpIX)-conjugated gold NPs to overcome several drawbacks, such as the low solubility and aggregation associated with PpIX. In vitro tests on HeLa cells highlighted gold NPs as effective PDT agents, evidenced by a significant reduction in cell viability at all tested concentrations [79].

Recently, one of the most studied aspects of gold NPs has been their optical characteristics. The main reason behind this is the recent advances in biomedical research, in which the optical properties of gold NPs are frequently highlighted. Moreover, the strong LSPR of the gold NPs plays an important role in this field by leading to improvements in diagnostic and therapeutic technologies. Hence, considering both previous and ongoing research regarding gold NPs, understanding and optimizing these attributes in a detailed manner would be crucial for advancing their applications across various fields.

### 2.5. Electrical Conductivity

Metals are recognized as remarkable electrical conductivity agents due to their unique atomic structures [80]. The presence of free-moving electrons on their outer membranes leads to the formation of a conduction pathway, where electrical currents can pass through with minimal resistance [81]. This characteristic is also applied at the nanoscale, in which metal NPs are widely employed as conductive additives in electronics, catalysis, and sensing studies [82,83,84]. Among metal NPs, gold NPs stand out with their outstanding electrical conductivity [85]. Their high efficiency as doping and coating materials has led to significant advances in current research by increasing the overall outcome of the electric conduction process [86,87]. As an example, Tommalieh et al. utilized gold NPs, prepared by the laser ablation method, in order to enhance the electrical properties of a Polyvinyl Pyrrolidone (PVP)/Polyvinyl Alcohol (PVA) blend. Confirming the successful distribution of NPs within the composite, they have evaluated the effect of gold NPs, with varying ratios, on the electrical conductivity performance. Findings highlighted an improvement in the AC conductivity of gold NP-doped PVP/PVA blends, especially when the NP concentration is increased [88]. In a similar way, Baei et al. developed an alternative strategy to address the electrical property limitations of implanted heart grafts, which are used for the regeneration of infarcted hearts. Through the experiments, they demonstrated an enhancement following the incorporation of gold NPs into chitosan hydrogels, compared to the samples containing chitosan alone. It was also emphasized that the concentration of the NPs holds significance, highlighted by a 1.9-fold increase in electrical conductivity when different concentrations of gold NPs (36 and 72 mM) are used [89]. Further, it is important to mention gold NPs’ wide utilization in the industrial area. Their superior electrical conductivity compared to other metal NPs has positioned them as one of the primary preferences when developing inks for electronics. In addition, gold NPs’ high stability, along with their resistance to oxidation and corrosion, is considered advantageous for the fabrication of conductive inks [90]. However, despite their potential, the high cost of gold NPs emerges as a limitation that hinders their applicability for mass production [91]. Lastly, gold NPs’ electrical conductivity can be influenced by their physical properties. Multiple studies in the current literature have shown that the electrical conduction efficiency of gold NPs is inversely proportional to their size [92], Hence, their synthesis methods and characterization must be tailored carefully, especially for applications requiring electrical conductivity, along with the requirement for further research.

### 2.6. Thermal Conductivity

Apart from their remarkable electrical characteristics, gold NPs exhibit high thermal conductivity, arising from the efficient transfer of heat by the movement of both phonons and electrons [93]. Hence, they have been widely employed in various areas, including in electronics, energy systems, and biomedicine [94]. To be more specific, gold NP-incorporated electronic cooling, solar desalination, and thermal management systems, in which the efficiency of heat transfer and dissipation is crucial, are being developed [95].

Similarly, the integration of gold NPs into composite nanofluids is considered an effective approach, where researchers take advantage of gold NPs’ stability and high surface area to enhance thermal conductivity [96].

Furthermore, the thermal properties of gold NPs can be affected by their physical attributes. As an example, Essajai et al. investigated the influence of gold NP shapes on the thermal conductivity of nanofluids. Focusing on gold nanospheres and NRs, they have revealed that rod-shaped gold NPs were more effective in improving nanofluid thermal conductivity in comparison to their spherical counterparts. These results were attributed to several factors, such as the solid–liquid interface, enhanced mobility of liquid atoms in nanofluids, and surface effects [97]. However, contradicting results were achieved when the size of the NPs was considered. Shalkevich et al. evaluated the impact of size on the gold NPs’ ability to transfer heat. Synthesizing spherical NPs with sizes varying from 2 to 45 nm, they have measured the conductivity of nanofluids with concentrations of 0.00025-1 vol%. The results indicated a negligible enhancement in thermal conductivity, with only 40 nm sized gold NPs showing 1.4% improvement [98].

In brief, the superior thermal conductivity of gold NPs highlights them as promising materials for various uses. However, a full understanding of the factors that influence their efficiency is required to maximize their potential.

### 2.7. Delivery

**Figure 4:**
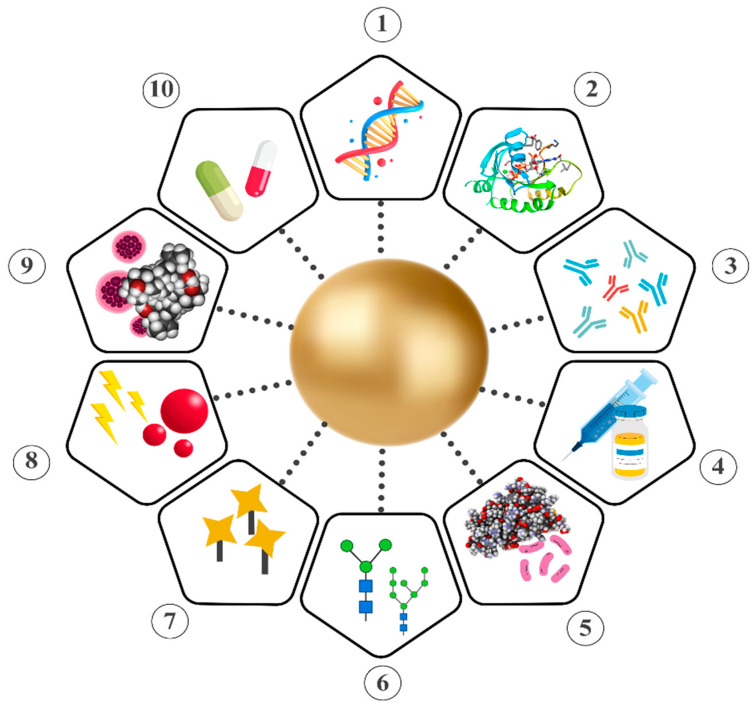
Functionalized gold NPs can be utilized for the delivery of a wide range of molecules, including nucleic acids (1), proteins (2), antibodies (3), vaccine adjuvants (4), antibacterials (5), glycans (6), imaging probes (7), photosensitizers (8), chemotherapeutic agents (9), and drugs (10) [7].

Gold NPs are considered promising carriers for therapeutics due to their facile synthesis, ease of functionalization, tunable physical properties, and low toxicity [99]. They are frequently used for the delivery of biomolecules, including proteins, nucleic acids, and chemotherapeutic agents, since they provide an effective approach for delivery systems [100] (Figure 4).

The size of gold NPs can be tuned for specific applications to enhance overall efficiency and facilitate deeper penetration into target tissues. For instance, research has focused on the synthesis of smaller gold NPs to overcome the blood–brain barrier (BBB), which is regarded as one of the biggest obstacles when developing future therapies where the direct delivery of functional drugs to the brain is desired [101]. On the other hand, modifying the surface of gold NPs with various ligands or functional groups, such as therapeutic agents, amino acids, proteins, peptides, oligonucleotides, and DNA, not only enhances their interaction with their surroundings but also enables more precise targeting. Therefore, researchers are developing delivery systems with enhanced sensitivity and specificity [102].

Moreover, gold NPs are known as highly biocompatible and low-toxicity materials, which is mostly attributed to their intrinsic properties, including chemical inertness, non-reactivity, and non-immunogenicity [103]. This makes them one of the safest materials for delivery systems, especially with their capability of not harming healthy tissue while delivering drugs to the target area.

*Protein delivery*

Proteins are one of the most abundantly found organic molecules within cells. Consisting of one or multiple chains of amino acids, they serve as essential components for both cellular integrity and activity. In addition, they play significant roles in building and repairing tissues, supporting immune function, and producing the enzymes and hormones necessary for biological processes [104]. Gold NPs present an efficient platform for the delivery of proteins by mitigating certain drawbacks. Some of these drawbacks include poor solubility through cell membranes and instability against digestive enzymes [105]. Proteins can be conjugated to gold NPs using various approaches, either with covalent or noncovalent bonding. Covalent bonding, primarily utilizing thiols, serves as a basis for chemical attachment with the molecular cargo. Amines are also used for covalent bonding, although they possess a lower bond strength relative to thiols [106]. On the other hand, non-covalent attachments include electrostatic and hydrophobic interactions, where the balance of these two is regarded as the key to achieving maximum binding affinity [107]. Non-covalent bonding is also notable due to being reversible, which allows the protein cargo to be easily released from the NPs under certain conditions [108].

Recently, gold NPs have been employed as efficient carriers for peptides, regarded as the building blocks of proteins. Farhangi et al. synthesized laminin peptide-decorated gold NPs, with a spherical shape and a size of 9.75 ± 2.40 nm. Their aim was to target and repair MS lesions through the administration of laminin. In vivo experiments on mouse models revealed a significant accumulation of peptide-modified NPs in the lesion area, with gold content reaching up to 4.675 ± 0.56 μg/g of brain tissue in contrast to 1.073 ± 0.66 μg/g for the control group [109]. Similarly, Liu et al. focused on the delivery of the peptide CopA3 along with ginsenoside CK, which has been known to exhibit antimicrobial effects (including antibacterial and anti-inflammatory characteristics) and anticancer properties. Synthesizing spherical gold NPs, ranging in diameter between 10 to 30 nm, researchers aimed to assess the anti-inflammatory properties of bioconjugated NPs. Modified gold NPs suppressed the expression of pro-inflammatory cytokines, such as IL-6, iNOs, COX-2, TNF-α, and IL-1β, considerably reducing their levels by 37.0%, 34.7%, 51.9%, 24.7%, and 33.6% following pre-treatment. It was also stated that NPs significantly alleviated ROS levels, with concentrations of 20 µg/mL and 40 µg/mL, leading to the inhibition of ROS production by 40.4% and 65.05%, respectively [110].

*Nucleic acid delivery*

Delivery of nucleic acids is considered crucial for the development of therapeutic approaches in the treatment of various diseases, including genetic disorders and cancers [111]. However certain limitations, such as toxicity, rapid degradation, poor transfection efficiency, and lack of targeting, hinder their utilization on a broader scale [112]. Hence, gold NPs come forward as highly stable, less toxic, and biologically inert inorganic nanomaterials, as they can significantly improve the overall efficiency of the delivery process [113]. Guo et al. developed siRNA–gold NP complexes by using the electrostatic interaction between the positively charged amino groups on the surface of the gold NPs and negatively charged siRNA. Using positively charged spherical gold NPs, ranging in size between 2 and 200 nm, the capacity of gold NPs in the delivery of nucleic acids was evaluated. It was stated that gold NPs were similarly efficient to traditionally used vectors; thus, they can be used in the creation of novel approaches for the delivery of genetic cargo [114] Similarly, Mout et al. utilized gold NPs in the CRISPR/Cas9 system, which has been one of the main focuses of the latest genetic research due to its potential to treat human genetic diseases. By co-assembling gold NPs with a single guide RNA and Cas9 protein, the efficiency of the vectors in delivering nucleic acids and proteins to the cytoplasm was assessed. Results revealed approximately 90% delivery efficiency in various cell types, along with up to 30% gene editing efficiency [115]. Another similar study delivered the SARS-CoV-2 DNA vaccine through chitosan–gold nanostars [116]. The DNA vaccine was delivered through intranasal administration in mice and induced significant antibody levels without a significant reduction for weeks. The induced levels of antibodies increased IgG and IgA levels by a noticeable amount.

Recently, Li et al. synthesized gold NPs to deliver siRNA against glucose transporter protein-1 (Glut1). Glut1 plays a critical role in the progression of cancer by enhancing glucose transport and is also known to be associated with aggressive tumor behavior when overexpressed. Their findings revealed successful inhibition of human lung cancer cell proliferation (A549) and in vivo xenograft tumor growth after the administration of a gold NP complex, achieved through the knockdown of Glut1 that limits glucose uptake [117]. DNA conjugation is also used in anticancer activity, which was evaluated in research that used DNA aptamers to detect cancer cells and destroy them [118].

Based on these findings, it can be concluded that gold NPs possess a wide array of uses in the delivery of nucleic acids, including gene delivery, gene editing, and cancer research. Hence, they can be regarded as efficient platforms for future approaches, owing to their facile synthesis, ease of functionalization, and high biocompatibility.

*Chemotherapeutic agent delivery*

Gold NPs are considered highly effective carriers for the targeted delivery of chemotherapeutic agents since they possess advantageous characteristics including low toxicity, inertness, high drug-loading capacity, and the ability to be easily functionalized with a broad range of organic molecules [119]. The mechanism of release of the chemotherapeutic agents from the gold NPs can depend on either internal or external stimuli, such as pH and light, respectively [7].

As an example, Khutale et al. synthesized spherical gold NPs, ranging from 20 to 25 nm, for the development of a pH-responsive system concerning the delivery of the chemotherapeutic agent Doxorubicin (Dox). In this system, researchers utilized a polyamidoamine (PAMAM) G4 dendrimer, which facilitated the attachment of Dox through amide bonds. In vitro studies revealed approximately 50% Dox release over 96 h at pH 4.0 (in comparison to pH 7.0), attributed to the cleavage of the amide bond between Dox and the dendrimer in acidic conditions [120]. Similarly, Joshi et al. conjugated gold NPs (with an approximate size of 7 nm) with chloroquine, an antimalarial drug that was recently discovered to have anticancer properties. *Through* in vitro experiments on MCF-7 cell lines, they have revealed the pH-dependent release of chloroquine, accompanied by an enhancement in cytotoxicity compared to the unmodified NPs. More specifically, approximately 62% of the drug was released at pH 6.0 after 48 h, which is an increase of up to 81% compared to lower pH conditions in the same period [121].

On the other hand, Niikura et al. focused on the photoresponsive release of Dox from cross-linked gold NP vesicles. Following incubation of Dox-encapsulated gold NPs with HeLa cells for 2 h, a diode laser at 532 nm was applied for 5 min to induce Dox release. After laser irradiation, a significant increase in the ratio of dead HeLa cells was observed compared to the samples without laser activation, confirming the effectiveness of the drug delivery system [122].

Moreover, the overall stability of the payload can be improved through functionalization with gold NPs. Kalimuthu et al. showed that pegylated gold NPs provide a more stable and less toxic environment for peptide–drug conjugates (PDCs), materials that are being developed to deliver anticancer drugs but that are often hindered by their low stability and short half-life in biological fluids. Through in vitro experiments, researchers showed a considerable increase in the half-lives of gold NP-coated PCDs, extending from 10.6 to 15.4 min to 21.0 to 22.3 h [123].

*Glycan delivery*

In addition to proteins, nucleic acids, and chemotherapeutic agents, glycans are another class of biomolecules that are employed by researchers for delivery by gold NPs. Glycans are important carbohydrate segments. In nature, glycans can either be free or bound to other structures, including proteins, lipids, or peptides. They are essential for a number of cellular processes involved in both health and illness. By participating in cell adhesion and receptor activation, they defend the host from viral and microbial invasions. Protein folding, conformation, solubility, immunogenicity, antigenicity, and resistance to proteolysis are all strongly influenced by glycans. The ability of glycans to form the microbial community of the developing gastrointestinal tract and act as a source of prebiotics is one of their most well-studied roles [124].

In recent years, the use of glycans with NPs has been an emerging topic. Researchers are particularly focused on developing vaccine adjuvants, especially in the area of oncology, by taking advantage of the glycans’ advantageous properties. As an example, Parry et al. synthesized gold NPs, with sizes ranging from 5 to 20 nm, decorated with Tn antigens. A Tn antigen is a mucin-type *O*-glycan whose expression is correlated with cancer and various human disorders and is regarded as a promising candidate for the development of immunotherapies due to its strong interaction with tumors [125]. Immunological assays showed that these NPs generated long-lasting and strong immune responses, shown by higher levels of antibody titers (IgG), in comparison to the samples delivered without NP conjugation [126]. Recently, Thomas-Moore et al. used gold NPs as carriers for glycans in PDT. Researchers modified 16 nm gold NPs with glycan-functionalized polyacrylamide probes such as galactose, glucose, lactose, and mannose. These glycan-modified gold NPs were then tested for their ability to inhibit the proliferation of breast cancer cell lines (SK-BR-3 and MDA-MB-231). Findings revealed that gold NPs selectively targeted cancerous cells through glycan–lectin interactions, with galactose-functionalized NPs showing the highest uptake [127].

From another perspective, glycans are also combined with gold NPs to treat infections caused by various pathogens. Since multivalent glycan–lectin interactions are leveraged by pathogens to bind and infect host cells, researchers can create surfaces that resemble pathogen structures by attaching glycans to gold NPs [128]. This mimicry creates a collective arrangement of glycans, similar to a glycocalyx structure that covers the cell surface, and enhances lectin binding, as well as increasing stability, solubility, and cost-effectiveness [129]. In this manner, Kulkarni et al. utilized glycan-encapsulated spherical gold NPs, with an approximate diameter of 4 nm, for the inhibition of Shiga toxins (Stx1 and Stx2) released from *Shigella dysentriae* and *E. coli* O157:H7. These toxins are known to be the main reason for various lethal disorders, such as hemolytic uremic syndrome, that may cause severe damage to the human body. Conducting experiments on luc2P Vero cells, they have observed a significant improvement in ED_50_ values from 0.07 ng/mL to 25.6 ng/mL (for Stx1) and 0.6 ng/mL to 92 ng/mL (for Stx2). This indicated the effective inhibition of Shiga toxins, requiring 366 and 153 times more Stx1 and Stx2 to inhibit protein synthesis, respectively, in the presence of glycan-conjugated NPs [129].

Given the synergistic effect of gold NPs with proteins, nucleic acids, chemotherapeutic drugs, and glycans, it is possible to develop novel drug delivery systems that would contribute significantly to the area of biomedicine. Hence, focusing on crucial parameters, such as optimization, delivery efficiency, and biocompatibility, will be crucial for expanding the utility of gold NPs in future therapeutic strategies.

### 2.8. Anticancer Activity

Gold NPs’ extraordinary characteristics, such as inherent stability, resistance to oxidation, ease of surface functionalization, biocompatibility, and strong optical traits, position them as ideal candidates for a broad array of biomedical uses [75]. In addition to their wide utilization in imaging, sensing, and delivery systems, their employment is increasingly expanding, especially in the design and development of novel anticancer agents [59]. Gold NP-based anticancer studies demonstrate low toxicity toward normal cell lines, whereas other commonly used metals, such as platinum [130] and silver NPs [131], tend to induce significant toxicity at high concentrations. In cases where high dosing is required, gold NPs present a notable advantage over other metal NPs. In a study, Babaei et al. focused on the anticancer efficiency of spherical gold NPs, with an average diameter of 17 nm, on human glioblastoma U-87 and U-251 cell lines. Following 72 h of exposure, it was stated that gold NPs triggered the induction of autophagy and significantly inhibited the proliferation of cancer cells [132]. Similarly, Safwat et al. synthesized spherical gold NPs, with sizes ranging from 9 to 17 nm, to enhance the anticancer effect of the antimetabolite drug 5-Fluorouracil (5-FU) on colorectal cancer cells. Findings revealed a significant improvement in its anticancer effect, with functionalized gold NPs reaching up to a 2-fold increase in effectiveness compared to 5-FU alone [133].

From another point of view, green synthesis methods have become highly popular in nanomaterial research, which has also been applied to gold NPs for the development of eco-friendly and novel systems to mitigate toxicity [134]. Padalia et al. used green synthesis to synthesize gold NPs using the aqueous leaf extract of *Ziziphus nummularia*. Spherical NPs with an average size of 11.65 nm showed dose-dependent in vitro toxic effects on T-47D, HeLa, and human fibroblast normal cell lines. When the concentration of gold NPs increased from 2 to 200 μg/mL, the percent cell viability of T-47D, HeLa, and human fibroblast cells decreased from 100 to 44%, 94 to 39%, and 100 to 50%, respectively [135]. In another study, Babu et al. employed the marine seaweed *Acanthophora spicifera* for the green synthesis of gold NPs. Spherical-shaped NPs, with a size less than 20 nm, exhibited cytotoxicity towards the human colon adenocarcinoma (HT-29) cell line, with a half-maximal inhibitory concentration (IC_50_) of 21.86 µg/mL [136]. Considering both chemical and nature-friendly approaches, Virmani et al. conducted a comparative study on the anticancer potential of gold NPs synthesized through different methods. Comparing chemically synthesized (spherical, hexagonal, and oval) and green-synthesized (spherical) NPs from *Ocimum tenuiflorum*, they have revealed superior anticancer properties through biological synthesis. Cell viability assays on the HeLa cell line indicated that treatment with biologically synthesized gold NPs at a concentration of 200 µg/mL was able to decrease cell viability to 50%. On the contrary, chemically synthesized NPs showed 80% viability even at a higher concentration of 400 µg/mL [137].

Overall, it can be concluded that gold NPs hold promising potential in cancer treatment, especially considering the recent advances in widely utilized green synthesis approaches. Further improvements in these methods will not only enable less expensive and nature-friendly production but also contribute to the development of novel anticancer agents utilizing nanomaterials such as gold NPs.

### 2.9. Antibacterial Activity

NPs have emerged as novel, cost-effective, and highly stable tools in antibacterial research, showing significant efficiency even against antibiotic-resistant strains of bacteria [138]. Among these, silver NPs have come forward as the most studied nanomaterials, as they exhibit high antibacterial activity towards a wide range of pathogens, with diverse mechanisms involving the constant release of silver ions [22]. Since gold NPs do not include such an effective mechanism, they are regarded as less powerful antibacterial agents than silver NPs. Still, thanks to their tunable surface characteristics, these NPs offer significant modification possibilities that can enhance their antibacterial activity to notable levels [139]. Thus, multiple papers in the current literature highlight gold NPs, usually combined with various molecules, as effective antibacterials. Lee et al. showed that gold NPs demonstrated antibacterial activity against *E. coli*, with a minimum inhibitory concentration (MIC) value of 16 μg/mL, without increasing ROS levels. Gold NPs, with a diameter of 30 nm, inhibited cell growth through the induction of membrane depolarization, overexpression of caspases, such as proteins, and DNA fragmentation, which collectively resulted in an apoptosis-like cellular death pathway [140]. From another perspective, Piktel et al. assessed the effect of shape on the antibacterial activity of gold NPs. Following the synthesis of rod-, star-, spherical-like-, and peanut-shaped NPs, antibacterial efficiency was tested on *E. coli*, *S. aureus*, and *P. aeruginosa*. Findings revealed ROS-mediated potent bactericidal activity, followed by outer and inner membrane permeabilization, with non-spherical-shaped gold NPs [141]. Additionally, Radhi et al. synthesized spherical gold NPs using a one-step pulsed laser ablation method then evaluated their bactericidal efficiency against four different bacterial strains. Gold NPs with a concentration of 1250 µg/mL showed high activity against *P. aeruginosa* and *S. aureus*, while *Acinetobacter Baumannii* (*A.baumannii*) was strongly affected even at 1000 µg/mL. In addition, *Streptococcus mutans* showed sensitivity towards all tested concentrations [142].

Researchers are also trying to maximize gold NPs’ potential by developing unique approaches through surface functionalization with various molecules, such as antibiotics and peptides, or by employing unique green synthesis methods [143]. For example, Hagbani et al. synthesized cefotaxime-loaded spherical gold NPs. In vitro tests highlighted the potent antibacterial activity of these NPs against both Gram-positive and Gram-negative strains, with MIC values down to 0.68 µg/mL, 0.73 µg/mL, 0.87 µg/mL, and 1.03 µg/mL for *S. aureus*, *E. coli*, *P. aeruginosa*, and *Klebsiella oxytoca*, respectively [144]. Similarly, Jana et al. showed that gold NPs conjugated with virstatin can be employed as alternatives to antibiotics, since multidrug resistance is becoming a major problem. Spherical NPs with an average diameter of 17 nm effectively inhibited the proliferation of *Vibrio cholerae* (*V. cholerae*), with an IC_50_ value of 0.1 nM [145].

On the other hand, there is a growing interest in green synthesis methods, as they provide nature-friendly and cost-effective alternatives to traditional production steps. Elias et al. used *Melaleuca cajuputi* leaf extract to synthesize gold NPs. Spherical gold NPs, with sizes ranging from 10 to 50 nm, caused bactericidal effects on *Vibrio parahaemolyticus*, showing MIC and minimum bactericidal concentration (MBC) values of 0.0075 g/mL [146]. In addition, Kerdtoob et al. recently developed a novel approach, where gold NPs are synthesized from Gram-positive *Streptomyces monashensis* MSK03. Biosynthesized gold NPs, possessing an average particle size of 23.2 nm and a spherical shape, were tested on drug-resistant *A. baumannii* and *P. aeruginosa*. Accordingly, antibacterial activity was observed with measured inhibition zones of 9.44 (±0.80) mm for *A. baumannii* and 11.20 (±0.67) mm for *P. aeruginosa* [147].

### 2.10. Antioxidant Activity

Oxidative stress is caused by an imbalance in the production of ROS. It results in a dangerous state where cellular damage occurs [148]. Hence, the requirement of antioxidant systems to alleviate harmful effects, such as the disturbance of cellular integrity, is significant. Metallic NPs (including gold) have attracted attention as promising antioxidants for their strong catalytic activity in radical scavenging reactions [149]. This is mainly due to the ability of gold NPs to adsorb molecules and scavenge free radicals on their surface [150]. Researchers also highlighted a possible interaction between the conduction electrons of the gold NPs and unpaired electrons from the free radical, improving the overall antioxidant efficiency [151]. In addition, the inherent properties of gold NPs contribute to their utilization in this research area, since they demonstrate chemical inertness, high resistance to oxidation, and biocompatibility [152]. In line with this, researchers highlighted ligand-modified gold NPs as superior antioxidants in comparison to their platinum and free ligand counterparts [153]. In addition, there are studies in the current literature underlining the comparable antioxidant activity of gold NPs with that of silver NPs [154]. Considering these, gold NPs can be considered potent candidates for the development of safer and more biocompatible approaches in further research.

Recently, it has been shown that gold NPs synthesized by green synthesis methods have significant antioxidant capabilities [134]. By using various quantities of NPs, researchers assessed the antioxidant capability of gold NPs derived from oak gum in terms of their ability to scavenge DPPH radicals. As a positive control, we employed butylated hydroxytoluene, and we contrasted the results of scavenging activity with the potential of oak gum. For oak gum and NPs, the scavenging activity was 54.56% and 21.54%, respectively [155]. The antioxidant potential of gold NPs produced from *Curcuma pseudomontana* (*C. pseudomontana*) was also examined by researchers employing DPPH scavenging activity, H_2_O_2_, reducing power, nitric oxide, and the CUPRAC assay, with ascorbic acid serving as the standard material. Results demonstrated a considerable correlation between the quantity of NPs and their capacity to stifle the activity of free radicals. Gold NPs also have superior scavenging capabilities compared to ordinary materials [156]. It has been discovered that the aqueous flower extract of Achillea biebersteinii functions as a capping/reducing factor in gold NP biosynthesis, which is a quick, safe, and economical procedure by Mobaraki et al. The superoxide radical reduction abilities of the extract and Ab-gold NPs were excellent and comparable to the typical antioxidant rutin [157]. Another recent study employing an aqueous extract of *Allium sativum L.* leaf showed that gold NPs had similar antioxidant activity and high potential for DPPH inhibition, with an IC_50_ value of 231 μg/mL [158].

These investigations have demonstrated the superior scavenging performance of gold NPs and highlighted their use as promising antioxidants in further research [159].

Research over the last five years has demonstrated the remarkable qualities of gold nanoparticles (Gold NPs) in a number of domains, such as drug delivery systems, environmental assessments, and biological applications, as indicated in Table 1.

**Table 1:** Research highlighting the properties and applications of Gold NPs in the past five years (2019–2024).

Highlighted Activity	Synthesis Method	Property	Result	Ref
Electrical conductivity	Chemical reduction	Size = 10 nm Shape = Spherical	Gold NP-decorated porous carbon microspheres were developed as electrode materials for supercapacitors to enhance electrochemical performance.	[160]
Electrical conductivity	Electrodeposition	Size = Ranging from 20 to 30 nm Shape = -	Gold NPs were incorporated into amperometric sensors to enhance their sensitivity and electrochemical performance.	[161]
Electrical conductivity	Chemical reduction	Size = Ranging from 1 to 6 nm. Shape = Spherical	Incorporation of gold NPs into supercapacitor dielectric composites enhanced electrical conductivity and specific capacitance.	[162]
Electrical conductivity	NPs were purchased commercially	Size = - Shape = -	Enhancement in the electrical conductivity and electrochemical properties of gelatin methacrylate hydrogels were observed following incorporation of gold NPs.	[163]
Electrical conductivity	Turkevich method	Size = Ranging from 9 to 46 nm Shape = Spherical	Gold NPs (combined with copper nanowires) enhanced electrochemical conductivity in amperometric sensors, achieving up to a 2.3-fold increase in performance.	[164]
Electrical conductivity	Laser ablation	Size = Ranging from 19.43 nm and 32.76 nm. Shape = -	Gold NPs increased the electrical conductivity of the PVP/PVA matrix, with higher concentrations leading to greater AC values.	[88]
Electrical conductivity	Seeded growth	Size = Ranging from 15 nm to 80 nm for spherical NPs. Length of 40.4 nm and width of 12.0 nm for rod-shaped NPs. Shape = Spherical and Rod-shaped.	Addition of gold NPs enhanced the fluid’s conductivity, with smaller spherical NPs improving electrical properties more effectively than their counterparts.	[165]
Electrical conductivity	Green synthesis from plant extract Laser ablation	Size = Ranging from 3 nm to 24 nm for green synthesized NPs; 2 nm to 30 nm for those synthesized through laser ablation. Shape = triangular, hexagonal, spherical and irregular	Incorporation of gold NPs enhanced the electrical properties of the polymer blend. Increased AC and DC conductivity, along with improved dielectric permittivity, was observed.	[166]
Electrical conductivity	Electrodeposition	Size = - Shape = -	Increase in electron transfer was observed following modification of carbon electrodes with gold NPs.	[167]
Electrical conductivity	Laser ablation	Size = Average size of 77 ± 4 nm. Shape = Spherical	Addition of Gold NPs into cement-based composites enhanced electrical conductivity, decreased electrical resistance, and increased the piezoelectric response by up to 57 times.	[168]
Thermal conductivity	Laser ablation	Size = Average diameter of 6.3 nm Shape = Crystalline structure	Incorporation of gold NPs improved the thermal conductivity of the nanofluid. Achieving 0.41 W/mK, a 26% increase compared to the base fluid was observed.	[169]
Thermal conductivity	Chemical reduction	Size = Ranging from 20 to 40 nm. Shape = Spherical	Incorporation of gold NPs into silica gel composites enhanced thermal conductivity by approximately 10–15%.	[170]
Thermal conductivity	NPs were purchased commercially	Size = Having a diameter of 41 nm Shape = Rod-shaped	Gold NPs improved the thermal properties of tissue-mimicking phantoms by increasing temperature response during photothermal therapy.	[171]
Thermal conductivity	Chemical reduction	Size = - Shape = -	Incorporation of gold NPs improved the thermal conductivity of carbon nanotube fibers by 70%.	[172]
Thermal conductivity	-	Size = Having approximate diameter of 4 nm Shape -	Gold NPs enhanced heat transfer in the tree-structured polymer networks by increasing the number of thermal transfer channels.	[173]
Optical Properties	Seeded growth	Size = Length of 45 nm and 65 nm Shape = Rod-shaped	Highly active SERS substrates were developed using hollow gold–silver NRs.	[174]
Optical Properties	Chemical reduction	Size = Having a diameter of 13 nm Shape = -	An optofluidic biosensor was developed using DNA-functionalized gold NPs for detection of mutated β-thalassemia sequences.	[175]
Optical Properties	NPs were purchased commercially.	Size = Having a diameter of 50 nm. Shape = Rod-shaped.	Gold NPs were developed to enhance photothermal performance in localized tumor treatment.	[176]
Optical Properties	NPs were purchased commercially	Size = Average size of 95.74 nm Shape = Spherical	Colorimetric biosensor utilizing gold NPs was developed for the enzyme-free detection of *Klebsiella pneumoniae* (*K. pneumoniae*).	[177]
Optical Properties	Turkevich method	Size = Having an approximate diameter around 40 nm Shape = Spherical	Electrochemical sensor utilizing gold NPs was developed for the detection of catechol.	[178]
Optical Properties	Turkevich method	Size = 14 nm Shape = Spherical	Gold NP-based lateral flow immunoassay was developed for the detection of tuberculosis antigens CFP-10 and ESAT-6.	[179]
Optical Properties	NPs were purchased commercially	Size = 40 nm Shape =	Gold NP-based electrochemical immunosensors were developed for the detection of HER-1 and HER-2 biomarkers in breast cancer.	[180]
Optical Properties	Chemical reduction	Size = Average size of 40 nm Shape =	Gold NP-integrated plasmonic biosensors were developed for the early detection of Familial Mediterranean Fever.	[181]
Optical Properties	Chemical reduction	Size = Average size of 53.88 ± 1.81 nm Shape = Spherical	Gadolinium-functionalized gold NPs were developed for dual-modal imaging and photothermal therapy in tumors.	[182]
Optical Properties	Chemical reduction	Size = Average diameter of 16 ± 1 nm Shape = Spherical	Gold NPs were developed for the rapid detection of microRNAs in milk samples to assess milk quality and cattle health.	[183]
Delivery	Chemical reduction	Size = Ranging from 5 nm to 20 nm Shape = Spherical	Targeted drug delivery system against SARS-CoV-2 was developed.	[184]
Delivery	Seeded-growth	Size = Average hydrodynamic size of 98.6 ± 0.6 nm Shape = Urchin-like	Nasal drug delivery system utilizing gold nanourchins as a carrier for targeted brain delivery was developed.	[185]
Delivery	Chemical reduction	Size = 2 nm. Shape = Spherical	Delivery system using ultra-small gold NPs to cross the BBB was developed.	[186]
Delivery	Chemical reduction	Size = Ranging from 13 nm to 18 nm Shape = Spherical	Non-viral gene delivery system utilizing gold NPs as carriers for hepatocellular carcinoma treatment was developed.	[187]
Delivery	Turkevich method	Size = Average diameter of 12 nm Shape = Spherical	Delivery system using gold NPs for the controlled release of dacarbazine was developed.	[188]
Delivery	Chemical reduction	Size = Average diameter of 35 nm Shape = Spherical	Resveratrol-gold NP delivery system to inhibit cataract formation was developed.	[189]
Delivery	Brust-Schiffrin method	Size = 4 nm. Shape = -	Delivery system using gold NPs, functionalized with cRGD peptides, for the delivery of the anticancer drug DM1 was developed.	[190]
Delivery	Gold NPs, with a concentration of 3000 ppm, were purchased commercially	Size = Having a diameter of 10 nm Shape = -	Gold NP–photosensitizer conjugates were developed to enhance the efficiency of PDT in targeting lung cancer stem cells.	[191]
Delivery	Chemical reduction	Size = Average diameter of 24 nm Shape = -	Gold NP-conjugated MRI contrast agents were developed to enhance the specificity and sensitivity of MRI imaging.	[192]
Delivery	Chemical reduction	Size = Average diameter of 13 nm Shape = -	Gold NPs were developed for the effective delivery of miR-206 to reduce cell viability and induce apoptosis in breast cancer cells.	[193]
Delivery	Chemical synthesis	Size = Average diameter of 63.3 ± 2.33 nm Shape = Spherical (Gold shell-iron core NPs)	Significant improvement in the encapsulation efficiency of curcumin by 81 ± 1.43% and drug loading capacity by 19.32 ± 0.6%. In vivo mouse model showed the anticancer activity of gold NP-based curcumin delivery with reduced tumor growth and volume. Increased IFN-γ (nearly 2-fold) and decreased IL-4 levels (nearly 4-fold).	[194]
Delivery	Chemical reduction	Size = Approximately 1.5 nm Shape = -	Chemotherapeutic drug oxaliplatin (OX)-loaded gold NPs achieved tumor inhibitory efficiency of 80.1% in a mouse model, surpassing that of the 44.6% and 61.6% by low and high concentrations of free OX, respectively. Drug-loaded gold NPs did not cause hepatotoxicity or hepatorenal toxicity, even at higher doses, and demonstrated a higher safety profile than the treatments utilizing OX alone.	[195]
Anticancer activity	Chemical reduction	Size = Average size of 90.6 (±9.6) nm Shape = Rod-shaped	Anticancer agents using gold NPs decorated with bovine serum albumin were developed.	[196]
Anticancer activity	Green synthesis using licorice root extract	Size = 2.647 nm to 16.25 nm size range Shape = Spherical	At high concentrations, the gold Np mediated by licorice root demonstrated superior antiproliferative action against MCF-7.	[197]
Anticancer activity	Green synthesis using marine microbe *Vibrio alginolyticus (V. alginolyticus)*	Size = 100–150 nm Shape =	With a maximum cell death inhibition of 25 mg/mL, the biosynthesized gold NPs showed a dose-dependent inhibitory effect on colon cancer cell growth.	[198]
Anticancer activity	Green synthesis using *Fusarium solani*	Size = 40–45 nm Shape = Needle and spindle like shape	On MCF-7 and HeLa cells, these gold NPs had strong cytotoxic effects.	[199]
Anticancer activity	Green synthesis using *Trachyspermum ammi*	Size = Average 16.63 nm Shape = Spherical and spheroidal	HepG2 cancer cell lines were shown to respond favorably to these NPs as anticancer agents. The synthesized NPs’ ability to suppress biofilm formation against pathogens, *Listeria monocytogenes* and *Serratia marcescens (S. marcescens)*, at SUB-MICs.	[200]
Anticancer activity	Green synthesis using *Mangifera indica*	Size = 20 nm Shape = round, triangle, and irregular shape	Modest antibacterial, cytotoxic, and dose-dependent antioxidant activities were demonstrated by gold NPs.	[201]
Anticancer activity	Green synthesis using *Vicoa indica* leaf extract	Size = Average size of 13 nm Shape = Spherical	Anticancer activity against a lung cancer cell line (A549), with an IC_50_ value of 73.56 µg/mL, was observed.	[202]
Anticancer activity	Green synthesis using *Gelidium pusillum*	Size = Average diameter of 12 ± 4.2 nm Shape = Spherical	Gold NPs demonstrated anticancer activity against cancerous cells (MDA-MB-23), supported by an IC_50_ value of 43.09 ± 1.6 µg/mL.	[203]
Anticancer activity	Green synthesis using *Schizophyllum commune*	Size = Average size of 90 nm Shape = Spherical	Gold NPs demonstrated dose-dependent anticancer activity against A549 lung cancer cells. Increasing the doses, from 15 μg/mL to 25 μg/mL, led to decreased cell viability.	[204]
Anticancer activity	Green synthesis using *Cyclopia genistoides* leaf extract	Size = Average size of 37 nm Shape = Spherical and pentagonal	Dose-dependent anticancer activity was observed against PC-3, Caco-2, and MCF-7 cells. PC-3 cell death increased by 2.5-fold compared to MCF-7 cells at a concentration of 100 µg/mL of gold NPs.	[205]
Anticancer Activity	Green synthesis using endophytic Cladosporium species from *Commiphora wightii*	Size = Average size of 5–10 nm Shape = Spherical	Green-synthesized gold NPs did not induce any toxicity in an in vivo mouse model (concentration of 2000 mg/kg). Significant reduction in tumor cell volume from 11 ± 0.5 mL to 5.33 ± 0.2 mL. Increased lifespan of mice by 85%. Protective action of gold NPs on the hematopoietic system by increasing hemoglobin, lymphocytes, and monocytes, and decreasing neutrophil levels near to control group levels.	[206]
Anticancer Activity	Chemical Synthesis	Size = Average size of 49 nm Shape = -	Gold NP-incorporated nanogels enhanced the efficiency of radiation therapy through reducing tumor growth in an in vivo mouse model. Gold NP injection did not alter the body weight of mice and showed activity at lower doses without significant toxicity.	[207]
Antimicrobial activity	Green synthesis using Presley leaf, *Petroselinum crispum* (*P. crispum*), extract	Size = Ranging from 20 to 80 nm Shape = Multi-shaped and spherical	Gold NPs(A) (2.5 mL extract used) demonstrated antibacterial inhibition against two Gram-negative pathogenic bacteria and demonstrated the highest anticancer efficiency against human colon cancer cells (HCT116).	[208]
Antimicrobial activity	Green synthesis using *Mentha longifolia* (*M. longifolia*) leaves extracts	Size = 3.45 ± 2 nm Shape = Round oval	The NPs markedly enhance antibacterial, antioxidant, antinociceptive, analgesic, and sedative actions.	[209]
Antimicrobial activity	Green synthesis using *Citrus macroptera* (*C. macroptera*)	Size = 20 nm Shape = Pseudo-spherical	The gold NPs that were manufactured demonstrate antibiofilm action against a *P. aeruginosa* biofilm. Additionally, they primarily show cytotoxic effects on HepG2.	[210]
Antimicrobial activity	Green synthesis using *Cynodon dactylon* L. Pers (*C. dactylon*)	Size = 21–33 nm Shape = Spherical and irregular	Gold NPs exhibited significant antibacterial efficacy against pathogenic bacteria such as *Enterobacter cloacae*, *Staphylococcus haemolyticus*, *Staphylococcus petrasii* subsp. *pragensis*, and *Bacillus cereus*, with inhibition zones of between 12 and 13 mm.	[211]
Antimicrobial activity	Green synthesis using *Scutellaria baicalensis*	Size = 20–40 nm Shape = Spherical	Gold NPs had strong cytotoxic, antibacterial, and antioxidant properties. They were not hazardous to RAW 264.7 or A549 cells, according to in vitro cytotoxicity data.	[212]
Antimicrobial activity	Green synthesis using *Jatropha integerrima* (*J. integerrima*)	Size = 38.8 nm Shape = Spherical	Maximum and minimum antibacterial activity against *B. subtilis* and *E. coli* is demonstrated by the gold NPs. *B. subtilis*, *S. aureus*, *E. coli*, and *K. pneumoniae* were shown to have MICs of 5.0, 10, 2.5, and 2.5 lg/mL, respectively, when gold NPs were used.	[213]
Antimicrobial activity	Green synthesis using *Platycodon grandiflorum*	Size = 15 nm Shape = Spherical	The *P. grandiflorum* gold NPs that were produced demonstrated effective antibacterial action against *B. subtilis* (11 mm) and *E. coli* (16 mm).	[214]
Antimicrobial activity	Green synthesis using *Arthrospira platensis* extract	Size = Average size of 10.98 nm Shape = Rod-shaped	Antibacterial activity against *Streptococcus pneumoniae* was observed with an MIC value of 12 μg/mL.	[215]
Antimicrobial activity	Green synthesis using *Lysinibacillus odysseyi* PBCW2	Size = Average size of 31.6 ± 9.7 nm Shape = Spherical	Antibacterial activity was observed against both Gram-positive and Gram-negative strains (*S. aureus*, *E. coli*, *V. cholerae Shigella dysenteriae*, *Aeromonas hydrophila*, and *Salmonella typhi*). MIC and MBC values were found to be between 25 to 40 μg/mL and 60–85 μg/mL, respectively.	[216]
Antimicrobial activity	Seeded-growth	Size = 82.57 nm Shape = Rod shaped	Gold NPs demonstrated antibacterial and antifungal activity against *E. coli*, *S. aureus*, and *Candida albicans* (*C. albicans*), at concentrations ranging from 0.25 ng/mL to 0.125 ng/mL.	[217]
Wound Healing Activity	Green synthesis from aqueous extract of *Acalypha indica*	Size = 20 nm Shape = Rod and Spherical	An in vivo mice model showed the improved re-epithelialization of the tissue with gold NP treatment, slightly on day 2 and significantly on day 7. Complete epithelialization on day 15. In vitro antibacterial and antioxidant activities were also evaluated.	[218]
Wound Healing Activity	Green synthesis from seeds of *Cydonia oblonga*	Size = Average size of 74 ± 4.57 nm Shape = Cubic and rectangular-shaped	Gold NPs induced significant wound healing capability in an in vivo mouse model. After 5 days, gold NP treatment nearly reduced the wound diameter by 90%, 6-fold higher than the control group. Significant in vitro antibacterial activity.	[219]
Antioxidant activity	Green synthesis using Oak gum extract	Size = Average 10–15 nm Shape = crystalline structure	It was found that the material demonstrated remarkable antioxidant properties through DPPH radical scavenging experiments.	[155]
Antioxidant activity	Green synthesis using *C. pseudomontana* isolated curcumin	Size = Average 20 nm Shape = Spherical	Effective antibacterial, anti-inflammatory, and antioxidant properties were exhibited by the gold NPs.	[156]
Antioxidant activity	Green synthesis using *Achillea bieber-steinii* flower extract	Size = Average 8 nm Shape = Spherical	It was discovered that the Ab-gold NPs were efficient against DPPH radicals. In addition, they showed better DPPH scavenging action than the plant extract did.	[157]
Antioxidant activity	Green synthesis using *Paracoccus haeundaensis* BC74171^T^	Size = Average size of 20.93 ± 3.46 Shape = Spherical	Antioxidant activity was observed, with a DPPH radical scavenging percentage ranging from 13.04 ± 3.14% at 10 μg/mL to 73.04 ± 3.01% at 320 μg/mL.	[220]
Antioxidant activity	Green synthesis using *Vitex negundo* (*V. negundo*) leaf extract	Size = Ranging from 20 to 70 nm Shape = Spherical	DPPH radical scavenging activity reached 84.64% at a concentration of 120 µg/mL, along with an IC_50_ value of 62.18 µg. Nitric oxide assay indicated 69.79% scavenging activity with an IC_50_ value of 70.45 µg, for the same tested concentrations.	[221]
Antioxidant activity	Green synthesis using *Hubertia ambavilla* plant extract	Size = Average size of 50 nm Shape = Flower-shaped	DPPH radicals were neutralized with an IC_50_ value of 16.5 μg/mL. Dose-dependent reduction in UV-A-induced MMP-1 production in normal human dermal fibroblast cells was observed, achieving an IC_50_ of 9.25 μg/mL.	[222]
Antioxidant activity	Green synthesis using *Glaucium flavum* leaf extract	Size = Average size of 32 nm Shape = Spherical	DPPH assay revealed a dose-dependent antioxidant effect of gold NPs. At concentrations of 125 μg/mL, 500 μg/mL, and 1000 μg/mL, the NPs achieved reductions in DPPH radicals of 23%, 37%, and 44%, respectively.	[223]
Antioxidant activity	Green synthesis using *Capsicum annum* fruit extract	Size = Ranging from 20 to 30 nm Shape = Spherical	DPPH assay showed 86% efficiency of NPs, at a concentration of 100 µg/mL, in comparison to Vitamin C that displayed 69.3% efficiency for the same tested concentrations.	[224]
Antioxidant activity	Green synthesis using *Nostoc calcicola*	Size = Ranging from 20 to 140 nm Shape = Triangular, spherical and cuboidal	DPPH radicals were effectively neutralized, with an IC_50_ value of 55.97 μg/mL.	[225]
Antioxidant activity	Green synthesis using curcumin isolated from *C. pseudomontana*	Size = Average diameter of 20 nm Shape = Spherical	DPPH, hydrogen peroxide, nitric oxide, reducing power, and CUPRAC assays showed dose-dependent antioxidant activity. At the highest concentration of 25 μg/mL, NPs exhibited inhibition rates of 85.2%, 83.2%, 84.5%, 87.9%, and 85.6%, respectively.	[156]
Bone Regeneration	Chemical Reduction Method	Size = Average diameter of 65.1 nm Shape = -	Gold NP-included scaffolds significant increased osteoblast and osteocyte concentration of rats from 23.25 ± 4.65 to 97.60 ± 27.16. Notable increase in fibroblast and fibrocyte amounts from 62.10 ± 3.55 to 109.11 ± 22.45.	[226]

## 3. Synthesis of Gold Nanoparticles

**Figure 5:**
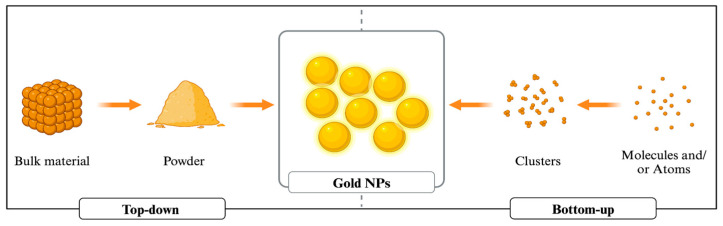
Top-down and bottom-up methodologies for the production of NPs [102].

There are two types of approaches used in the synthesis of gold NPs: top-down and bottom-up schemes. Top-down approaches entail the fragmentation of complex material into NPs, whereas bottom-up approaches begin at the atomic scale. Figure 5 depicts the fundamental procedures included in each method. Laser ablation [227], ion sputtering [228], UV and IR irradiation [227], and aerosol technology are examples of synthesis methods that use the top-down approach. On the other hand, the bottom-up approach involves the reduction of Au^3+^ to Au^0^.

Thus, based on the top-down and bottom-up mechanisms, the production of gold NPs encompasses a range of techniques, such as chemical, physical, and green/biological procedures, which will be further explained below.

### 3.1. Physical Synthesis

The γ-irradiation approach has been demonstrated to be the most effective for synthesizing gold NPs with adjustable dimensions and high purity. The γ-irradiation technique is employed to produce gold NPs ranging from 5 to 40 nm in size. This approach employs a natural polysaccharide alginate solution as a stabilizer [229].

Gold NPs are produced by a photochemical process employing chloroauric acid (HAuCl_4_) and an aqueous glycine solution subjected to UV irradiation. The initiator comprises amino acid-capped gold NPs that are subsequently functionalized with glycine [230].

Using cetyltrimethylammonium bromide (CTAB) as a binding agent and citric acid as a reducing agent, the microwave irradiation method was used to produce gold NPs [231]. Gold NPs are manufactured using either the heating approach or the photochemical reduction method. Citrate, tartrate, and malate ligands were employed for lowering HAuCl_4_ [232]. A photochemical methodology has been documented for the synthesis of gold-polyethylene glycol core-shell NPs, measuring 10–50 nm in aqueous solution, utilizing redox and polymerization processes. This approach involved the reduction of gold salt through radical production, utilizing polyethylene glycol diacrylate via a UV process facilitated by a photo-initiator, 2-Hydroxy-2-methyl-1-phenyl-1-propane [233].

Laser ablation synthesis in solution is a straightforward process for the fabrication of NPs from various solvents. The irradiation of various metals submerged in a solution by a laser beam generates a plasma that produces NPs. The top-down methodology for metal reduction to NPs, employing laser ablation techniques, provides a stable solution devoid of stabilizing agents or chemicals [234]. Laser ablation in an aqueous biocompatible media can be used to produce gold NPs, which significantly alters the surface chemistry as well as the physical and biological characteristics [235].

Because of their distinct surface chemistry, laser-synthesized NPs can react with a range of innovative biocompatible materials [236]. For example, they may have O− functionalization at a high pH due to the presence of some higher oxidation states, which enables hydrogen bonding interactions. Without any group ligand-specific binding, gold NPs can be non-covalently conjugated to a wide range of substances, enhancing their potential as innovative biocompatible materials for unexplored or underdeveloped biological applications [237].

In order to prevent contamination by certain undesirable compounds that can obstruct biological research, gold NPs have been generated and functionalized utilizing the capabilities and benefits of fs laser ablation by Correard et al. Their findings demonstrated that stable and monodisperse gold NPs may be produced using laser ablation in solutions containing dextran, polyethylene glycol, and water [238]. The formation of gold NPs by the pulsed laser ablation of bulk, high-purity gold in deionized water was examined in a similar study. The scientists irradiated the gold surface with a Q-switched Nd:YAG laser, then looked at how the thickness of the liquid layer and the post-ablative modification processes affected the size, aggregation, and ablation effectiveness of the gold NPs. The average particle size was found to be decreased from 15.12 nm to 9.5 nm by laser-induced NP modification, and the size distribution was narrowed with 532 nm pulses. This allowed for the control of NP redistribution and average size in ablated colloid solutions. Without the need for any additional chemical reagents, Wender et al. produced stable gold NPs by simply ablating an Au foil that was positioned inside or outside of four ionic liquids (ILs) using a laser. Following laser ablation, irregularly shaped spherical gold NPs with a diameter ranging from 5 to 20 nm were created. The produced NPs’ size and form were found to be correlated with the location of NP nucleation and growth, which might be either within or at the IL surface. In fact, the stabilization of the gold NPs created by laser ablation outside of the ILs was significantly influenced by the surface ion orientation and chemical makeup of the IL/air interface [239].

Vacuum sputtering is a method employed in the fabrication of thin films or coatings, such as gold NPs. This method relies on the application of a potential difference between the two electrodes within the vacuum chamber, creating an electric field. An inert gas enters the chamber and undergoes ionization. A metal target (cathode) is bombarded with argon plasma. As a result, atomic clusters are extracted from the target location and deposited onto a surface or into a liquid solution [240]. ILs make it possible to prepare the metal NP system, which lowers the need for successive stabilizers. In order to prepare solutions via cathode sputtering, low vapor pressure is required. With the groundbreaking study of the Kuwabata group in 2006, the relatively new field of research on the creation of NPs by the sputtering method onto ILs was launched. Sputtering 1-ethyl-3-methylimidazolium tetrafluoroborate (EMI.BF_4_) directly onto its surface produced gold NPs with a diameter of 5.5 nm and a deviation of 0.9 nm [241]. By using plasma sputtering in a solution medium in an open system at atmospheric pressure, Hu et al. were able to create gold clusters (average particle size about 1.5 nm) and NPs (with an average particle size of approximately 3.5 nm) with spherical morphology. The solvent medium and temperature have an impact on the particle sizes. They showed that gold NPs of varying sizes might find use in a variety of industries [242].

A straightforward mechanical technique called ball milling uses attrition to create NPs. It moves kinetic energy from the reduction material to the grinding medium. Consolidation and compaction, an industrial-scale procedure where NPs are “put back together”, yield enhanced characteristics [22].

### 3.2. Chemical Synthesis

#### 3.2.1. Turkevich Method

The Turkevich method, initially documented in 1951, is a well-acknowledged approach for producing spherical gold NPs in the size range of 10–30 nm, therefore establishing it as the pioneering chemical system for the synthesis of gold NPs. This technique entails the reduction of gold precursors by the use of reducing agents such as trisodium citrate, ascorbic acid, amino acid, and polymers. The gold precursor is subjected to heat until it reaches boiling point, then a 1% solution of trisodium citrate is added while stirring vigorously. The formation of gold NPs is indicated by the color transition from pale yellow to wine red.

The Turkevich method is a systematic and consistent technique used to generate spherical particles within the size range of 10–30 nm. However, as particles exceed 30 nm in size, they lose their spherical shape and exhibit limited yield [243].

#### 3.2.2. Electrochemical Method

Using tetra alkyl ammonium salts as stabilizers in a nonaqueous solution, Reetz et al.’s 1994 work showed that NPs may be synthesized electrochemically, enabling the size-selective synthesis of transition metal particles [244,245]. Through the use of tetra alkyl ammonium salts as stabilizers of metal clusters in a nonaqueous solution, their research demonstrated that size-selective nano scales of transition metal particles may be configured electrochemically. The superiority of the electrochemical technique over alternative NP manufacturing methods has been confirmed, mostly attributed to its modest equipment requirements, low cost, reduced processing temperature, good quality, and facile yield control [246,247]. Using a basic two-electrode cell, the gold NPs were synthesized electrochemically by oxidizing the anode and reducing the cathode [248]. The electrochemical synthesis method can be used to produce gold NPs on the surface of multi-walled carbon nanotubes supported by glassy carbon electrodes [249].

#### 3.2.3. The Brust–Schiffrin Method

Brust–Schiffrin synthesis (BSS), initially presented in 1994, is a technique for synthesizing stable thiol-functionalized metal NPs, specifically designed for the preparation of gold NPs in organic solutions [250]. The procedure entails the immersion of a water-based gold precursor into an organic solvent, namely tetraoctylammonium bromide (TOAB), followed by its reduction with sodium borohydride (NaBH_4_) in the presence of alkanethiol. The transition in color from orange to brown signifies the synthesis of gold NPs [251].

The Brust approach is a straightforward technique for producing thermally and air-stable gold NPs with a precise size and dispersion. However, its drawbacks include producing less evenly distributed gold NPs and requiring the use of immiscible organic solvents [252].

#### 3.2.4. Seeded Growth Method

**Figure 6:**
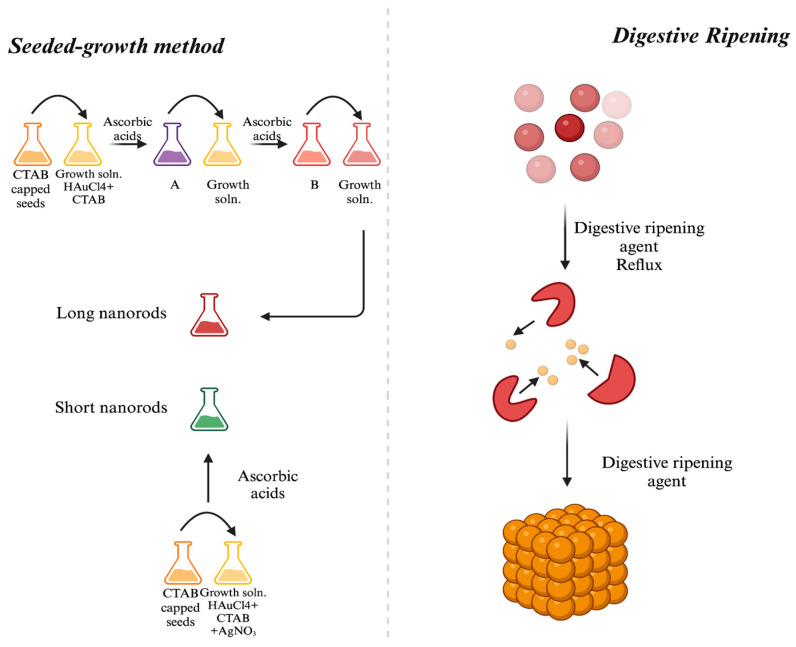
Steps in the manufacture of gold NPs. The seeded growth approach (A); The digestive ripening technique (B) [102].

Using the seeded growth technique, gold NPs can be produced in a range of geometries including rods, cubes, and tubes. This technique produces gold NPs of diminutive dimensions, measuring between 5 and 40 nm in diameter. It is straightforward, efficient, and inexpensive. This procedure entails the reduction of the precursor using a potent reducing agent (NaBH_4_) to generate seed particles. These particles are subsequently introduced into a metal solution that contains weak reducing agents (ascorbic acid or sodium citrate) to facilitate the formation of OH^-^ groups and structure-directed agents (Figure 6). Variations in the concentration of metal seed, reducing agents, and structure-directed agents can yield diverse nanostructures [251].

Effective synthesis of rod-shaped gold NPs by seed-mediated growth requires precise control of parameters such as the HAuCl_4_ concentration, temperature, and seed number. Greater concentrations of HAuCl_4_ led to the formation of larger seed rods with reduced aspect ratios, whereas elevated temperatures resulted in the formation of rods with lower aspect ratios [253].

#### 3.2.5. Digestive Ripening

An efficient approach to produce uniform gold NPs in the presence of an abundance of ligands (digestive ripening agents) is known as digestive ripening. The fundamental procedure involves subjecting a colloidal solution to elevated temperatures (about 138 °C) for 2 min, followed by additional heating at 110 °C for 5 h, utilizing alkanethiol. Thermal conditions are the primary determinant of the size distribution of gold colloids [254]. Furthermore, there are alternative techniques that provide the synthesis of gold NPs by the utilization of ultrasonic vibrations (Figure 6) [255,256].

A simple and useful chemical strategy for producing monodispersed NPs is the digestive ripening method. One other advantage of this approach is the substantial production of NPs [257]. An underlying drawback of the digestive ripening technique is the challenge of regulating the morphology of NPs when subjected to extremely high temperatures [258].

### 3.3. Green/Biological Synthesis

**Figure 7:**
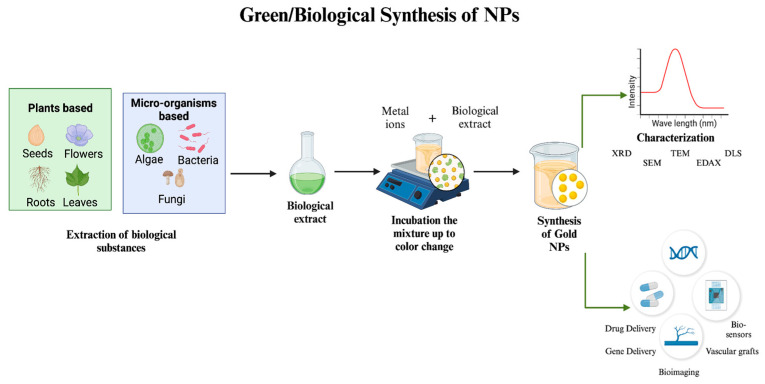
Graphical representation of the green/biological synthesis of gold NPs [260].

Although chemical and physical approaches are straightforward for rapidly producing gold NPs, their applicability in biology is limited by the requirement for toxic and expensive reducing agents or applications (Table 1) [259]. Hence, there is an increasing need to create environmentally sustainable and economical methods for the synthesis of NPs that do not involve the use of hazardous substances. NP synthesis using green methods has gained significant interest as an environmentally benign and sustainable approach in recent years. Green techniques involve the synthesis of NPs using microbes, enzymes, and plant compounds or plant extracts (Figure 7) [260].

#### 3.3.1. Microorganism-Based Gold NP Synthesis

Microorganisms, such as bacteria and fungi, are frequently employed in the manufacture of gold NPs. These microbes engage with organic metals and can produce gold NPs through both extracellular and intracellular mechanisms. Microorganisms are cultivated in settings with gold precursors, and subsequently extracted and purified by various procedures. The dimensions and morphology of gold NPs can be regulated by microbial growth factors [261].

When gold NPs interact with bases that include phosphorus or sulfur, they cause free radicals and break respiratory chains, which ultimately results in cell death. To further contribute to cell death, they might further decrease ATPase function and inhibit tRNA attachment to ribosomal subunits [262]. Gold NPs generate surplus electrons, exterminating Leishmania and creating ROS (O_2_ and OH). These radicals obliterate the pathogen’s DNA and other cellular constituents [263]. These gold NPs may also impede transmembrane H+ efflux, representing an additional possible mechanism [264]. Due to their diminutive size and approximately 250 times diminished antibacterial efficacy compared to bacterial cells, they are more prone to adhere to cell walls and impede processes that often lead to cell death [265]. Herdt et al. argued that interaction with a gold surface can damage DNA [266].

The precipitation of gold NPs in bacterial cells upon exposure to Au^+3^ ions was observed by Beveridge and Murray. Organic phosphate compounds contribute to the formation of octahedral gold, potentially serving as agents for biota–gold complexation. Both Fe^+3^-reducing bacteria and *Shewanella algae* (*S. algae* ) are capable of reducing Au^+3^ ions in anaerobic conditions [267]. *S. algae* and hydrogen gas fully decompose Au ions, resulting in the formation of gold NPs of 10–20 nm in size [268]. *V. alginolyticus* marine bacteria was employed by Shunmugam et al. to synthesize gold NPs. The highest suppression of colon cancer cell proliferation was seen at 25 mg/mL when the biosynthesized gold NPs were applied. The environmentally benign and economically viable green synthesis of gold NPs has anti-inflammatory and anti-cancer properties against colon cancer [198]. Unlike the other studies, gradient centrifugation was employed by Qui et al. to eliminate the hazardous components of gold NPs from *S. aureus*. Muscle cell viability was observed to benefit from the pure gold NPs, which also provided protection against damage caused by cardiotoxins. They showed promise in facilitating myocardial infarction healing by reducing infarct area and enhancing heart function when constructed onto an elastic scaffold to form a cardiac patch [269].

The incorporation of fungi in the synthesis of NPs is a relatively new development and shows potential for huge-scale NP manufacturing. Indeed, fungi release substantial quantities of the enzymes relevant to NP formation and are more easily cultivated in both laboratory and industrial settings. *Fusarium oxysporum* (*F. oxysporum*), *Verticillium* sp., *Thermomonospora* sp., and *Rhodococcus* sp. have been documented as fungal and actinomycete species capable of synthesizing NPs either intra- or extracellularly [270,271].

Because of fungi’s favorable growth characteristics, gold NPs may be produced on a huge scale in an industrial setting. Furthermore, fungi demonstrate high gold NP monodispersity. Yeast species including *Pichia jadinii* and *Yerrowiali polytica*, as well as fungal species including *F. oxysporum* and *Verticillium* sp., have a good ability to manufacture gold NPs [272].

The fungal manufacturing of gold NPs can occur both extracellularly and intracellularly. The internal mechanism can be elucidated by the reduction of sugars, proteins such as ATPase, glyceraldehyde-3-phosphate dehydrogenase, and 3-glucan-binding proteins implicated in the energy metabolism of fungal cells [273]. Au^+3^ permeates the cell membrane and is reduced by cytosolic redox mediators. It remains ambiguous whether the diffusion of Au^+3^ ions transpires across the membrane via active bioaccumulation or passive biosorption [274]. The research on fungal ultrathin slices revealed the concentration of gold NPs in the vacuoles of cells [275]. The extracellular synthesis of gold NPs transpires through the adsorption of AuCl4^−^ ions onto cell wall enzymes via electrostatic interactions with positively charged groups [276]. NADPH-dependent oxidoreductases, located either on the cell surface or within the cytoplasm, are essential enzymes in the biosynthesis of gold NPs, similar to their role in the synthesis of other NPs, such as silver NPs [277,278].

Algae, a distinctive source of chemicals such as fucoidan, neutral glucan, and alginic acid, have considerable medicinal importance owing to their antibacterial, anticoagulant, and antifouling properties. The production process can occur via both extracellular and intracellular pathways, rendering algae a unique subject for investigating the characteristics of gold NPs [279]. Blue-green algae, such as *Spirulina subsalsa*, which is a member of the Spirulinaceae family, have been used for manufacturing gold NPs. The green algae *Rhizoclonium hieroglyphicum* and *Rhizoclonium riparium* are members of the Cladophoraceae family. Chakraborty et al. [280] and Nayak et al. [281] have reported finding the diatoms *Nitzschia obtusa* and *Navicula minimum*. *Turbinaria conoides* alga extract was used to produce gold NPs in a green way. Color change and spectral data, such as a large SPR band at 520–525 nm, confirmed the synthesis of gold NPs. TEM and a crystalline structure with a size range of 6–10 nm were used to corroborate the structure of the produced Au [282]. In a similar study, *Tetraselmis suecica*, a marine microalga, was used as a reducing agent during the synthesis of gold NPs. The formation was verified by ultraviolet–visible (UV-vis) spectroscopy, which showed a peak at 530 nm. A Bragg reflection was detected using FTIR and X-ray diffraction (XRD) spectroscopy. The generation of well-dispersed gold NPs was confirmed by TEM and laser light scattering, with 79 nm being the most common particle size [283]. The optical, physical, chemical, and antibacterial characteristics of self-assembled gold NPs were revealed by their production, employing an extract from *Chlorella vulgaris* by Annamalai et al. From 2 to 10 nm in diameter, spherical self-assembled cores were seen in the gold NPs. They tested positive for *S. aureus* and *C. albicans* through biological screening, indicating that they may have use in green chemistry and as a strong medication [284]. Chakraborty et al. propose that secreted algal enzymes contribute to the biosynthesis of gold NPs, with NADPH-dependent reductase playing a pivotal role. This enzyme functions as an NADH electron carrier, effectively reducing Au ions to gold NPs via an enzymatically mediated electron transfer process within the inner mitochondrial membrane matrix [285]. An efficient, quick, and one-pot approach was used to achieve the synthesis of gold NPs utilizing *Cystoseira baccata* extract, a brown macroalga that is present in the Atlantic, Baltic, and Mediterranean Seas. Zeta potential measurement, TEM, high-resolution TEM, scanning electron microscopy (SEM), and UV–vis spectroscopy were used to analyze the biosynthesized gold NPs. To stop coalescence and aggregation, the extract was applied as a shielding substance [286]. *Tetraselmis kochinensis*, a green alga with a size range of 5–35 nm, has been used in the environmentally benign biosynthesis of gold NPs by Senapati et al. Because the cytoplasmic membrane and cell wall include enzymes that reduce metal ions, these gold NPs have a significant impact on the cell wall. They may find use in catalysis, biological applications, and medication administration [287].

In addition to complete organisms, biological products such as nucleic acids, amino acids, lipids, proteins (enzymes), and viroid capsules were utilized in the manufacture of gold NPs. These compounds contain carbonyl and hydroxyl functional groups, which facilitate the reduction of Au^+3^ ions to Au^0^ and are subsequently stabilized by stabilizing agents. Although numerous studies have documented the production of microbial gold NPs, the precise process remains inadequately defined and is now under investigation. According to the assumed process of microbial synthesis, the nucleation and development of gold NPs are caused by the enzymes released by microorganisms, which reduce Au^+3^ ions to Au^0^ [288]. The organic phosphate compounds may facilitate the formation of a complex with gold salts, leading to the in vitro accumulation of gold NPs in *B. subtilis* [289].

#### 3.3.2. Synthesis of Plant-, Fruit-, and Waste-Extract-Based Gold NPs 

When compared to microbial synthesis, plant-mediated inorganic NP synthesis has a number of benefits, such as the ability to create enormous quantities of NPs on an industrial scale and the avoidance of the labor-intensive maintenance of cell cultures [290]. Revaluing plant secondary metabolites based on polyphenols as effective reducing agents for metallic precursors is the mechanism-guided synthesis procedure. The hydroxyl groups present in plant-derived polyphenols were discovered to play a successful role in the reduction of gold ions by promoting the oxidation reaction and the particular synthesis of quinine forms [291].

Numerous research studies have documented the synthesis of gold NPs from plant leaf extracts. Leaf extracts of *C. pseudomontana* [156], *Lawsonia inermis* [292], and *Scutellaria barbata* [293] have been used to produce gold NPs. Antonio Zuorro’s study involved the synthesis of NPs from both the leaves and the juice extract of the plant. This study employed kiwi fruit juice to synthesize and stabilize gold NPs [294]. The juice of *C. macroptera* was successfully used to synthesize gold NPs. The NPs produced from *C. macroptera* juice extract exhibit an SPR band at 544 nm [210]. Gold NPs were produced by combining an appropriate quantity of *Punica Granatum* juice under mild reaction conditions in a separate study [295].

Furthermore, *Jasminum auriculatum* leaf extract, which functions as a stabilizing and reducing agent, is used in a trouble-free, ecologically friendly method for the biogenic manufacture of gold NPs as reported by Balasubramanian et al. The production of biogenic gold NPs was validated by the SPR peak in the UV–visible absorption spectra at 547 nm. The biogenic gold NPs have an average size of 8–37 nm and are spherical, according to TEM and SEM examinations. The biogenic gold NPs are a flexible choice for heterogeneous catalysis, as demonstrated by their catalytic reduction activity on p-nitrophenol. The biogenic gold NPs’ cytotoxicity demonstrated that the NPs significantly inhibited the proliferation of the human cervical cancer cell line at a dose-dependent level, with an IC_50_ value of 104 μg/mL [296]. In a different study, Al-Radadi attempted to generate innovative, environmentally friendly, and reasonably priced gold NPs using licorice root extract. On MCF-7 and HePG-2 (liver) cell lines, the cytotoxicity of green-produced gold NPs was also evaluated using an MTT technique. When tested against bacterial and fungal strains, as well as cell lines, gold NPs showed little antibacterial, antifungal, and reflected anticancer activities [197]. Ullah et al. also evaluated the in vitro antibacterial efficacy of gold NPs generated from the aqueous extract of *Tamarindus indica*, and they examined the in vivo sedative and analgesic properties of the crude extract. The samples exhibited significant antibacterial efficacy against *K. pneumoniae*, *B. subtilis*, and *Staphylococcus epidermidis*, demonstrating a 10–12 mm inhibition zone. Gold NPs exhibited stability under elevated temperatures, varying pH levels, and in a 1 mM saline solution. They demonstrated sedative and analgesic properties, underscoring the significance of phytochemical-mediated gold NP production [297]. The study by Ruf et al. examined the analogous biological activities of green-produced gold NPs, favoring *M. longifolia* leaf extracts for their manufacture. The dimensions and morphology of the NPs were validated using sophisticated techniques, revealing polydisperse and spherical configurations of gold NPs measuring 13.45 ± 2 nm. In comparison to MLE, the NPs markedly enhance antibacterial, antioxidant, antinociceptive, analgesic, and sedative actions [209]. El-Borady et al. utilized Presley leaf (*P. crispum*) extract for the first time to produce gold NPs, which were then employed as antioxidant, anticancer, antibacterial, and photocatalytic agents. The research employed four extract volumes to synthesize gold NPs, yielding four distinct sizes and morphologies. The NPs were evaluated using many approaches, with gold NPs(D) (20 mL plant extract) exhibiting the highest antioxidant capacity. The NPs exhibited antibacterial action against two Gram-negative bacteria, but not against Gram-positive bacteria. Photocatalytic efficacy for the degradation of methylene blue dye was attained rapidly [208]. Using seed extract from *Trachyspermum ammi*, the researchers created gold NPs for the study. They then investigated the NPs’ effectiveness against drug-resistant biofilms of *S. marcescens* and *L. monocytogenes*, as well as their potential to inhibit HepG2 cancer cell lines. Against these bacteria, the NPs demonstrated a strong biofilm inhibitory effect, blocking important components such as exopolysaccharides, motility, and CSH. Additionally, they reduced the amount of intracellular GSH in HepG2 cancer cells, which made them more susceptible to the production of ROS and led to apoptosis [200].

Human cervical cancer, breast cancer, and normal cell lines were used as test subjects for Mangifera indica gold NPs. With respect to concentration, cell viability varied, ranging from 98–67 to 98–60%. Gold NPs’ potential for anticancer activity is highlighted in this work [201]. The *J. integerrima* Jacq. flower extract was used to create gold NPs; Fast Fourier-Transform Infrared (FT-IR) revealed phenolic chemicals and the UV–vis spectrum revealed a high peak at 547 nm. Their crystalline nature was confirmed by XRD, SAED, TEM, and DLS (dynamic light scattering). The gold NPs had MICs of 5.0, 10, 2.5, and 2.5 lg/mL, respectively, against B. subtilis and E. coli. Their crystalline nature was proven by their spherical form and 38.8 nm size [213]. Similar to this, a straightforward and environmentally friendly method was used to create gold NPs that contained an aqueous extract of *C. dactylon*. When *C. dactylon*-loaded gold NPs were tested at varying concentrations (0.625–100 μg/mL) against the MCF-7 cell line, the IC_50_ was determined to be 31.34 μg/mL using the MTT assay [211].

This field of study is showing promise for the advancement of environmentally friendly nanomaterials, which could lead to the creation of sustainable solutions for pressing health and environmental issues. To fully realize this field’s potential and tackle difficult problems in a variety of industries, more research and development are needed.

### 3.4. Variables Influencing NP Synthesis

The manufacture, characterization, and applications of NPs are affected by several factors, including substrate selection and catalytic activity during synthesis. The dynamic characteristics of produced NPs change under various environmental conditions and influences. Critical parameters influencing NP formation encompass the solution’s pH, temperature, extract concentration, raw material concentration, particle size, and synthesis processes. This section delineates essential aspects affecting NP biogenesis, emphasizing the necessity of accounting for these factors in the manufacture, characterization, and application of NPs [298].

*pH*

The shape and size of NPs are significantly influenced by the pH levels of the substrate and the surrounding media. The charge and stability of the biomolecules in the extract can be affected by the pH of the solution, which can also change how they interact with gold ions and change the size and form of the NPs. Medium- to larger-sized NPs are usually produced in an acidic pH environment, and changes in the substratum’s pH cause the NPs’ size and form to change [299]. Polyakova et al. used citrus lemon to investigate how pH affected the synthesis of gold NPs. The findings demonstrated that whereas a basic pH (7–9) slows down the growth of NPs, an acidic pH (2.5–5) tends to promote NP growth by quickening the aggregation step. It was discovered by the researchers that the creation of 8 nm sized gold NPs can occur at a pH of 5 [300].

*Temperature*

Temperature substantially affects the formation kinetics of gold NPs and the mechanics of particle growth. Smaller NPs are the result of faster nucleation and growth at higher temperatures. With the right stabilizers, high temperatures and high salt concentrations can produce more anisotropic particles. Fluctuations in temperature factors influence the ultimate aspect ratio, length, and diameter of gold NRs. Reduced temperatures, between 21 and 35 °C, led to the formation of shorter and thinner gold NRs due to the energy necessary for particle growth [301]. The physical approach necessitates temperatures over 350 °C, whereas chemical procedures demand temperatures below 350 °C. Synthesis using green technology generally necessitates temperatures below 100 °C or ambient temperatures, as the temperature of the reaction medium affects the characteristics of the NPs [302]. According to a study by Mountrichas et al., the production of monodisperse gold NPs increases with temperature during the gold NP synthesis process [303]. Furthermore, another study found that gold NPs produced below 50 °C were highly polydisperse and that the lengthy reaction periods at low temperatures rendered the synthesis process impractical. For optimal and monodisperse nanoparticle production, a set high temperature (boiling temperature) and technique are therefore necessary [304].

*Pressure*

Pressure is crucial for NP production. The pressure exerted on the reaction medium influences the morphology and dimensions of the produced NPs. The rate of metal ion reduction utilizing biological agents is significantly accelerated at ambient pressure settings [305].

*Time*

Researchers investigated the impact of reaction time on gold NPs manufactured from palm leaves by extracting samples from the reaction media at intervals ranging from 5 to 480 min. The UV spectroscopy results indicated an increase in absorption wavelength from 5 min to 60 min. After 60 min, the wavelength stabilizes, indicating the complete reduction of gold ions [306]. Temporal differences may arise from numerous factors, including particle aggregation owing to prolonged storage, alterations in particle size with time, and inherent shelf life, all of which influence their potential [307].

*Concentration of source extract/biomass, salt, and reducing agents*

The efficacy of NPs is frequently determined by the concentration of the plant extract and salt solution. Several studies have observed the increased production of NPs, as well as changed forms. This is why it is frequently necessary to determine the right concentrations [308]. Variations in salt concentrations affecting NPs production have also been observed. The synthesis of NPs was enhanced by elevating the salt content, achieving optimal yield at a concentration of 0.7 mM [309].

On the other hand, several synthetic procedures and techniques have been developed to regulate the size, shape, and surface charge characteristics of gold NPs. Because of its great repeatability and control over particle size, synthesis based on citrate reduction is one of the many sophisticated techniques extensively employed in many applications; nonetheless, there are still important problems in this respect. The gold NPs are synthesized by the reduction of HAuCl_4_ by trisodium citrate dihydrate in aqueous solution. The findings demonstrate that the citrate concentration, utilized as a reducing agent and stabilizer, may substantially influence NP size, leading to stable gold NPs with consistent dimensions as a result of HAuCl_4_ reduction. The adsorption rate of the stabilizer governs the NP size during the reduction process [310].

### 3.5. Characterization of Gold NPs

**Figure 8:**
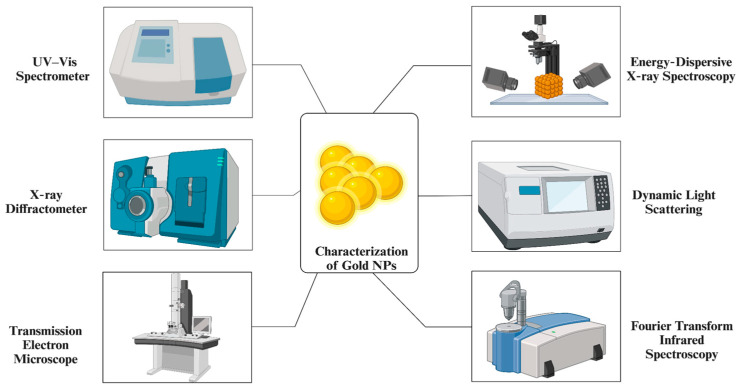
Characterization of Gold NPs [251].

Dimensions, surface arrangement, and morphology determine the physical characteristics of gold NPs. Characterization is key, which verifies the production of gold NPs with a color change from pale yellow to ruby red. Further characterization methods are employed to gain a deeper understanding of their characteristics (Figure 8) [251].

#### 3.5.1. Ultraviolet–Visible Spectroscopy (UV–Vis)

The size, concentration, and aggregation level of gold NPs may all be estimated using UV–vis spectroscopy, which is a very helpful method. UV–vis spectroscopy examination is a prevalent method for assessing the production of metal NPs by investigating their distinctive optical properties, which are contingent upon their size and morphology [311]. In the UV–vis spectrum, the gold NPs that were made show a clear single SPR band at 527 nm. In addition, most laboratories have UV–vis spectrometers; the analysis does not change the material, and spectrum registration takes little time. The extinction spectra of gold NP can be evaluated by Mie theory, contingent upon the modification of the metal’s dielectric constant to account for NP dimensions and physicochemical conditions, as evidenced by direct measurements on individual ensembles of NPs [312].

To offer a straightforward and rapid approach for assessing the size and concentration of gold NPs, Haiss et al. contrasted theoretical findings with experimental data. Gold NPs measuring between 5 and 100 nm were produced and analyzed using TEM and UV–vis. The dimensions and concentration of gold NPs can be directly ascertained from UV–vis spectra utilizing eqs 10^−13^ for the computation of d and eq 14 for the determination of N, together with the relevant fitting parameters [313]. The assessment of gold NP aggregation can be accomplished using UV–vis spectroscopic techniques. Indeed, when gold NPs combine, a shoulder appears at a wavelength of roughly 600 nm, which is near the distinctive SPR band. Overall, without sample pretreatment, this spectroscopic approach offers an effortless and quick analysis method for quality control right after their synthesis. For an accurate characterization of particle size, UV–VIS spectroscopy in conjunction with other analytical techniques is therefore essential [314].

#### 3.5.2. X-Ray Diffractometer (XRD)

The crystalline structure of gold NPs was determined by XRD research [315]. Using equipment that operated at a voltage of 20 mA and was effective at 40 kV, the sample preparation process entailed reducing the gold NP solution to be drop-coated on a glass surface using Cu Kα radiation [316]. The crystalline characteristics of gold NPs synthesized by *K. pneumoniae* were examined using XRD diffraction patterns, and the average size of these NPs was determined employing the Debye–Scherrer equation [317]. Four unique 2θ peaks at 38.1, 44.3, 64.1, and 77.7 are observed in gold NPs, which closely resemble the typical Bragg reflections of a face-centered cubic lattice. An intense diffraction peak at 38.1 suggests that Au0 has a preferential growth orientation in the 111 direction [318].

#### 3.5.3. Transmission Electron Microscope (TEM) and Scanning Electron Microscope (SEM)

While the optical microscope has a resolution limit of microns, electron microscopes such as a TEM can picture NPs on the nanoscale scale. TEM pictures are used to identify the size and surface morphology of NPs, making them appropriate for the structural and chemical characterization of nanomaterials at the nanoscale. TEMs can be magnified up to 50 million times to enhance the visibility of features [319]. On the other hand, the sizes and forms of gold NPs in the dry condition can be established using TEM imaging. It is possible to see that gold NPs have a distinct structural shape and accurate size measurements [320].

Furthermore, this approach requires sample preparation, which may lead to artifacts such as gold NP aggregation [314]. Surface alterations are undetectable by normal TEM techniques; specialized equipment, such as cryo-TEM, is necessary. Surface alterations are undetectable by ordinary TEM techniques; specialized equipment, such as cryo-TEM [321], glycerol spraying/low-angle rotating metal shadowing TEM [322], and others, is necessary.

SEM is a method that employs direct viewing to analyze NP morphology through electron microscopy. It provides benefits for morphological and dimensional analysis, although it also presents disadvantages, including insufficient data regarding size distribution and the actual population mean. The apparatus comprises an electron cannon, condenser lenses, and a vacuum system. SEM generates three primary types of images: external X-ray maps, backscattered electron images, and secondary electron images [323]. Preparing the sample for analysis with SEM involves coating copper grids with tiny layers of carbon. To produce these films, a small amount of material was dropped onto the grip, and the remaining solution was removed using blotting paper. The film was then further dried under a mercury lamp for a minimum of five minutes [324]. The average diameter of the gold NPs, which were discovered to be in a variety of geometries, including rectangle, square, cubic, and triangular, was 60 nm [317]. Notwithstanding these benefits, this technique is labor-intensive and expensive, frequently necessitating supplementary information regarding the size distribution.

#### 3.5.4. Energy-Dispersive X-Ray Spectroscopy (EDAX)

Energy Dispersive X-ray Spectroscopy (EDAX) is a radiological method employed to determine the fundamental composition of substances. Integrated with SEM/TEM, the microscope’s imaging capability facilitates specimen identification. Digital X-ray (EDX) data exhibit spectrum peaks, including the distinctive absorption band peak of metallic gold nanocrystallites in gold NPs, detected at around 2.2 keV [325].

#### 3.5.5. Dynamic Light Scattering (DLS)

Despite its long-standing history and popularity, people often arbitrarily select the weighting and mean of DLS, a prevalent method for assessing NP size, to conform to other techniques and expectations [326]. In contrast to other more complex methods, DLS is comparatively inexpensive and simple to use, making it the main instrumentation option for assessing the size and size distribution of NP suspensions [327]. More than 50% of drug products incorporating nanomaterials submitted to the US Food and Drug Administration’s Center for Drug Evaluation and Research in recent years used DLS to characterize size. Even though DLS measurements are widely used in the characterization of NPs, they nevertheless present a number of difficulties because of dubious data interpretation and processing [328].

Gold NPs exhibit exceptional light scattering properties at or near their SPR wavelength. Jans et al. preferred to use DLS as a highly practical and effective instrument for gold NP bioconjugation and biomolecular binding investigations. The conjugation of protein A with gold NPs under various experimental settings, together with the quality and stability of the resulting conjugates, was extensively monitored and evaluated using DLS [329]. Liu et al. employed DLS to enhance the understanding of potential interactions between gold NP materials and biomolecules both in vivo and in vitro, specifically focusing on gold NRs. In gold NRs with particular aspect ratios, the size distribution diagram displays two peaks: one at 70–80 nm and the other with a hydrodynamic diameter of 5–7 nm. Rotational diffusion produces the smaller peak, but when proteins adhere to the NRs, the peak associated with rotational diffusion significantly changes. They proved that DLS is a useful instrument for characterizing NRs. It provides information on gold NR–protein interactions supplementary to molecular spectroscopy methods [330].

#### 3.5.6. Fourier-Transform Infrared Spectroscopy (FT-IR)

For the identification of the functional group present between a reducing agent and a gold precursor in suspensions of NPs, FT-IR is a highly sensitive method. It validates the functionalization of gold NPs by illustrating the interactions between them and the reactive agent [331]. The utilization of contemporary computing tools enables the quantitative analysis of NPs to be accomplished in a few seconds.

#### 3.5.7. X-Ray Photoelectron Spectroscopy (XPS)

X-ray photoelectron spectroscopy (XPS) is an effective technique for analyzing surface properties (less than 10 nm) and is extensively utilized across various disciplines in science and engineering [332]. It is a quantitative method renowned for its superior capacity to ascertain chemical states, extensive applicability, and non-destructive characteristics. Nonetheless, it possesses deficiencies including inadequate spatial resolution (15 mm), moderate absolute sensitivity (0.01–1.00%), incapacity to identify hydrogen or helium, and protracted analytical durations. The deficiencies are progressively being rectified as the applications of the method expand. XPS applications encompass the analysis of surface functionality in organic and plastic coatings, the assessment of oxidation states in catalysts and nanomaterials, and the provision of both quantitative and qualitative data. These applications are compatible with diverse sample types, including gaseous, liquid, or solid, and are non-destructive [333]. XPS offers valuable insights into the coordination chemistry of ligands on NP surfaces, particularly in comparison to uncoordinated ligands [334].

#### 3.5.8. Thermogravimetric Analysis (TGA)

The physicochemical characterization of nanomaterials needs more analytical methods to figure out their shape and makeup, since their surface makeup can change when they come into contact with biological fluids or environmental factors. Precisely defining the surfaces and functional coatings on NPs is essential for achieving complete and uniform coverage during modification [335]. TGA is a dependable analytical method for evaluating the purity of nanomaterials by the observation of mass changes during heating. It generates a decomposition curve, indicating the oxidation temperature and remaining mass. The residual mass may result from inorganic leftovers, metal catalysts associated with synthesis, or contaminants. Nonetheless, TGA is both deleterious and costly. It can also evaluate organic residues, surface melting characteristics, and oxidation resistance [336].

## 4. Toxicity

Though their broad application depends on biosafety evaluation, gold NPs are interesting biomedical instruments. The need to assess their effects on health and learn more about their toxicity and biocompatibility is developing [337]. At first, the cytotoxic effects of novel nanomaterials are assessed since they may change the way cells function. Nevertheless, implicit biocompatibility is not always ensured by the absence of cytotoxicity. The basis for biocompatibility is how a material interacts with its biological surroundings; it guarantees that treated biomaterials will not produce any harmful or defensive reactions. This idea is essential to determining biocompatibility [338].

Biocompatibility is often defined as the ability of a certain substance or device to function well with living tissue or organisms. When a nanomaterial interacts with its host without causing harmful effects such as oxidative stress, DNA damage, mutagenesis, or apoptosis, biocompatibility is generally achieved. Prior to in vivo testing, cytotoxicity—the detrimental effect on a particular cell line—is usually evaluated using in vitro assays. Nevertheless, regardless of the methodology, existing studies on the toxicity of gold NPs are incongruous [339].

**Figure 9:**
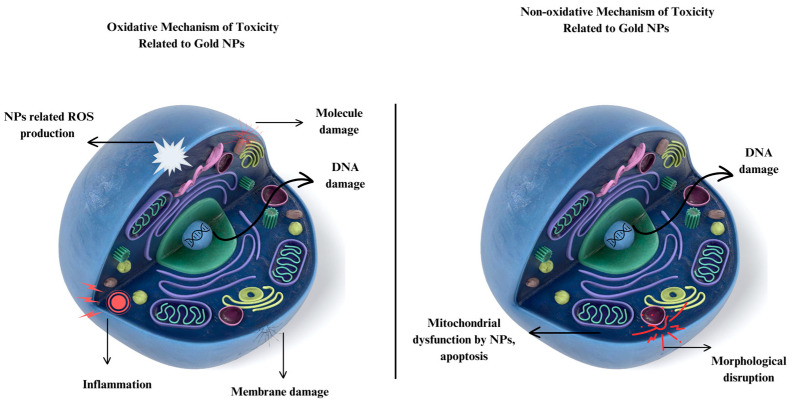
Graphical representation of the gold NP toxicity process (both oxidative and non-oxidative) [352].

Toxicity mechanisms of NPs are classified into two groups, including oxidative and non-oxidative mechanisms (Figure 9). One of the toxicity mechanisms of NPs is thought to be the production of ROS, which can inhibit antioxidants and cause oxidative stress, potentially resulting in inflammation and damage to molecules and cell membranes [340]. Additional oxidative stress can harm DNA, which triggers cell death processes in the cell [341]. Ozcicek et al.’s study demonstrated a correlation between elevated ROS generation and gold NP concentrations ranging from 1 μg/mL to 100 μg/mL [58].

In assessing the toxicity of gold NPs, one must consider various potential pathways that may be involved, including but not limited to genotoxicity, ROS generation, mitochondrial damage, cell death pathways, hazardous material leakage, the interaction of endocrine disruption with molecules, or alterations in cell morphology. Although oxidative stress is a well-known mechanism of NP toxicity, it is important to understand that there are a number of non-oxidative mechanisms through which gold NPs can cause toxicity. Due to its ability to disrupt multiple metabolic pathways and have an impact on amino acid synthesis, mitochondrial toxicity is the primary cause of gold NP cytotoxicity [342].

Since gold is naturally chemically inert and very unreactive, bulk gold is recognized to be harmless. Gold salts function as catalysts when they are present in molecular form. In contrast to bulk or molecular-scale gold, gold has several beneficial characteristics at the nanoscale, including easily programmable sizes, simple manufacture, simple modification, and potent optical qualities [343]. The existing research, both in vitro and in vivo, exhibits significant variability in methodologies and results. Numerous publications suggest that gold nanoparticles are harmless; nevertheless, other findings dispute this assertion. It is necessary to thoroughly assess the possible toxicities of innovative nanomaterials in biological systems both in vitro and in vivo while taking safety and biocompatibility concerns into account. Their negative consequences have drawn a lot of attention because of their many uses in biological domains [344]. Based on MTT assays, gold nanospheres of varying diameters (4, 12, and 18 nm) and capping agents (citrate, cysteine, glucose, biotin, and CTAB) were determined to be harmless using a human leukemia cell line [345]. When applied to immune system cell lines, gold NPs (spheres with a diameter of 3.5 nm) produced comparable outcomes [346]. Conversely, some research groups have determined that gold NPs exhibit “toxicity.” Goodman et al. discovered that cationic gold nanospheres, measuring 2 nm in diameter, exhibit toxicity at specific dosages. Notably, the NPs exhibiting a negatively charged surface were determined to be non-toxic in the same quantity and inside the same cell line. This observation was ascribed to the interaction of cationic NPs with the negatively charged cellular membrane, resulting in membrane breakdown [347]. In addition, 1.4 nm gold nanospheres caused necrosis, mitochondrial damage, and oxidative stress in every cell type they studied, according to Pan et al. Remarkably, they discovered no proof of cellular harm for gold nanospheres with the same surface group that were 15 nm in size. This finding raises the possibility of gold nanoparticle toxicity that varies with size [348].

Numerous journals have published reviews of NPs’ toxicity, primarily focusing on NP–cell/organism interactions within specific contexts (Table 2). Because high doses are employed in in vitro tests, which are not feasible for in vivo investigations due to the progressive clearance of NPs by renal and fecal excretion, in vitro and in vivo studies cannot be directly compared. Therefore, minimal dosages should be employed in in vitro tests for the assessment of nanotoxicity [349,350]. Furthermore, unlike with traditional chemicals or medications, the dosage–response connection cannot be ascertained by specifying the NP dose as a concentration. Other measurements, such as surface area or the quantity of NPs, are probably more relevant [351].

NPs show promise in biological applications; however, pharmacokinetics, biodistribution, and toxicity measurements require further focus, particularly when human subjects are involved. Nanomaterials ought to be both large enough to load particular components and small enough to penetrate biological barriers [353]. Because of their durability and capacity to target certain cells or tissues, gold NPs may find use in therapeutic settings. However, there are regulatory and safety issues with their broad usage, such as the possibility of toxicity from surface, size, and shape alterations, the absence of known routes for the metabolism and elimination of NPs, and the lack of evidence on the long-term impact on humans.

Humans are exposed to metal NPs through the bioapplication of nanomaterials; hence, a comprehensive evaluation of the health concerns associated with designed gold NPs is required. For preclinical studies of nanosafety and toxicity risk, this calls for the creation of new standardization and certification assays that assess physicochemical properties, sterility, pyrogenicity, biodistribution, ADME, pharmacokinetics, and in vivo and in vitro toxicity [354]. The ongoing efforts to standardize gold NP risk assessment methodologies still require refinement. While Standards Developing Organizations (SDOs) are working to produce key standards for nanomaterials, none have yet gained widespread acceptance. Nanomaterials are thought of similarly to conventional chemicals [103].

Requirements for compounds used in food, medicine, cosmetics, and devices have been established by US and European authorities. In the US, the FDA sets these criteria, although South Korea, China, and Japan have their own requirements for characterizing nanomaterials. Industries and regulatory environments in South American countries heavily rely on laws from the Brazilian Health Regulatory Agency (ANVISA). The types of nanomaterials and their intended uses must be taken into consideration when regulating their characterization, standards, and evaluation techniques [355].

When considering bioapplication, a systemic toxicity assessment of gold NPs is typically carried out using animal models, including mice or rats (Table 2) [356]. It is essential that gold NPs enter the body through the subcutaneous, intravenous, and oral routes. These particles have the ability to change or undergo metabolism in response to biological components. Because gold NPs are not specifically governed by current frameworks, it is difficult to guarantee quality and safety across batches. Preclinical testing, regulatory norms tailored to NPs, and moral human trials are necessary to address these issues [341].

Differently shaped gold NPs (spherical, rod-shaped, triangle-shaped, star-shaped, octahedron-shaped, plate-shaped, and prism-shaped) have been synthesized and their cytotoxicity has been evaluated. Steckiewicz et al. examined the cytotoxic effects of rod-shaped, star-shaped, and spherical gold NPs on human fetal osteoblast hFOB 1.19 and pancreatic duct cell hTERT-HPNE cell lines. Gold NP rods had the highest toxicity to human cells, whereas gold NP spheres demonstrated the lowest toxicity [357]. Wang et al. indicated that gold NP NRs exhibited significantly greater toxicity than gold NP hexapods. This emphasized that the cytotoxicity of gold NPs is contingent upon their form [358]. The arrangement of surface atoms in gold NPs may have been altered due to their varying geometries, particularly when comparing spherical shapes to star or rod configurations. A greater number of atoms at angles and edges may lead to enhanced interactions with biomolecules, resulting in toxicity in rods and stars, which is generally not seen in spherical NPs [359].

The immunological organs of broiler chickens were studied, and it was discovered that adding 15 ppm gold NPs to drinking water resulted in DNA fragmentation, elevated IL-6, histopathological alterations, oxidative damage, and a significant drop in antibody titer against avian influenza and Newcastle disease [360].

The suitability of gold NPs for surface modifications is crucial not only for controlling their toxicity but also for enhancing their effectiveness in imaging, diagnostic, and anticancer applications. Localized and specific agglomerations of gold NPs are key to the efficient detection or elimination of targeted structures. However, this also poses a major drawback in treatment areas, such as anticancer therapies and drug delivery, where agglomeration can present significant risks to human health [361]. To prevent off-target effects during gold NP treatment, their surface characteristics must be precisely controlled during the synthesis process. The surface charge of gold NPs significantly affects their biocompatibility, influencing uptake levels and potentially leading to non-specific binding [362]. The surface chemistry of gold NPs, modified with conjugated molecules such as PEG or chitosan, can also significantly influence their biodistribution [363].

**Table 2:** In vivo and In vitro Toxicity Studies of Gold NPs (2019–2024).

Type of Study	Organism	Particle	Effects	Ref
In vivo	Mice	Laser-ablated dextran-coated gold NPs	The absence of acute and chronic toxicities and healthy animal behavior supported the safety of gold NPs, which were mostly stored in the liver and spleen and did not produce hepatic or renal toxicity.	[364]
In vivo	Broiler chicken	Gold NPs	The therapy significantly damaged the blood’s oxidative capacity, altered histology, elevated the expression of the IL-6 and Nrf2 genes, fragmented DNA, and reduced the antibody titer against avian influenza and Newcastle disease.	[360]
In vivo	Rat	Gold NPs	Different doses of gold NPs had distinct hazardous effects on different organs. Non-toxic doses, on the other hand, had no effect on the testis and only mildly affected the liver and kidney.	[365]
In vivo	Zebrafish animal model	N-myristoyltaurine stabilized gold NPs	According to a toxicity study conducted on an animal model of zebrafish, gold NPs are safe.	[366]
In vivo	Rat	Gold NPs	Despite being non-toxic, the study discovered that greater doses of gold NPs, such as 2 mg/kg, were harmful to every organ examined.	[367]
In vitro	Human kidney-2 (HK-2) cell and proximal tubular cells	Gold NPs with different shapes (spheres and stars), capping (citrate and MUA), and diameters (13 nm and 60 nm)	For HK-2 cells, the 13 nm nanospheres were the most hazardous, damaging the mitochondria and lysosomes and increasing the generation of ROS. Severe MUA-capped gold NPs led to apoptosis. Larger 60 nm gold NPs considerably decreased cellular viability but were less hazardous.	[368]
In vitro	HK-2 and 786-0 cells	Gold NPs	Gold NPs with a diameter of 5 or 200 nm have the ability to trigger autophagy in HK-2 cells to shield them from harm and apoptosis in 786-0 cells to kill tumor cells.	[369]
In vitro	Keratinocyte cell line (HaCaT) and human epidermoid skin cancer cell line (A431)	*Vitis vinifera* (*V. vinifera*) gold NPs	*V. vinifera* seed gold NPs were non-toxic to normal HaCaT cells, but they suppressed the growth of A431 skin cancer cells by cytotoxicity and death.	[370]
In vitro	Cancer (Caco-2, MCF-7 and HepG2) and non-cancer (KMST-6) cell lines	* Terminalia mantaly * (TM) extract gold NPs	Using the MTT assay, the study investigated the cytotoxic effects of TM-gold NPs on cancer and non-cancer cell lines and discovered that certain extracts were more hazardous than others.	[371]
In vitro	Hep2 liver cancer cell line and Vero cell line	Gold NPs	The NPs exhibited remarkable non-toxic effects on the normal VERO cell line and anticancer activities in the treated Hep2 liver cancer cell line.	[372]
In vitro	HeLa cell lines	*V. negundo* extract gold NPs	At greater dosages, gold NPs are hazardous to HeLa cells.	[373]
In vitro	Human HepaRG cells or primary rat hepatocytes (PRH)	Gold NPs with different size (~15 nm and 60 nm), shape (nanospheres and nanostars) and capping [citrate- or 11-mercaptoundecanoic acid (MUA)],	In serum-free media, the 15 nm MUA-capped nanospheres exhibited considerable toxicity to PRH and HepaRG cells, indicating that their restricted application in diagnostics should be disregarded.	[374]

## 5. Future Trends

Since they are nontoxic and biocompatible, gold NPs have become increasingly important in many areas of nanotechnology. Green synthesis pathways should prove beneficial in places where traditional gold NPs are already having an impact. Because of their strong antibacterial action, plant-based gold NPs are anticipated to be useful in the fight against antimicrobial resistance. A new generation of broad-spectrum antimicrobial medications might be developed as a result of gold NPs; by 2021, the global gold NPs market is expected to increase at a compound annual growth rate of 18.84 percent. Gold NPs have a practical and affordable market through green/biological synthesis, especially for commercial uses such as point-of-care testing, large-scale diagnostics, vaccine research, and home use [375]. Human health has advanced significantly as a result of recent approvals and trials, including the use of gold NPs in nanomedicine. Standardized synthesis and characterization procedures are necessary for gold NP technologies in order to guarantee uniform quality throughout manufacturing batches. Standardized frameworks for assessing gold NPs’ toxicity, biocompatibility, and long-term stability might be established through cooperation between researchers, industry stakeholders, and regulatory agencies. Preclinical research and long-term safety assessments are required to address health issues and guarantee safety. Putting money into environmentally friendly production techniques can reduce environmental hazards and encourage wider adoption of nanoparticle technology. Clear regulatory paths can speed up approval procedures and preserve public confidence in drug or device frameworks. Thus, the international community needs to create particular protocols for the preclinical development and characterization of these products because of their distinct physicochemical and optical features and possible bioapplications.

One of the most prominent applications of gold NPs is in anticancer research. Not only are they efficient anticancer agents, but they can also be utilized in cancer imaging and drug delivery, enabling multiple approaches to anticancer research [30]. The need for advanced methodologies and novel agents in anticancer research is well-established. Gold NPs are among the most suitable metal NPs for surface modification. Given their application in anticancer research and their ability to be modified with antibodies, patient-specific anticancer drug delivery and treatments are expected to become a major trend in the near future [376]. Given the importance of alternative treatments in anticancer research and their low toxicity combined with green synthesis methods [377], gold NPs are expected to become one of the leading nanomaterials in future anticancer research.

Another unique characteristic of gold NPs is their capability in colorimetric assays for molecular detection and diagnostic applications. Various types of molecules, such as pharmaceutical compounds, heavy metals, and enzymes, can be detected with gold NP-based colorimetric sensors. Moreover, various agents found in foods, including chemicals, microorganisms, and antibiotics, can also be detected using colorimetric sensors [378]. Most importantly, the color change is detectable with the naked eye, up to a certain threshold, offering a significant advantage in sensing experiments. These wide-ranging possibilities for gold NP-based sensing applications are all due to their unique optical properties. Thanks to their significant SPR, many PTT-based applications show great promise in diagnostics.

Green synthesis, surface modification, and the excellent optical properties of gold NPs are also being applied to another emerging field: wastewater treatment and detection in environmental applications. Various gold NP-based sensors can be used for dye absorption, heavy metal detection, and the immobilization of chemical toxins [379]. However, the lack of research on gold NP toxicity and the absence of optimized large-scale production using green methods still hinder the development of these promising trends, especially in environmental applications [380].

**Figure 10:**
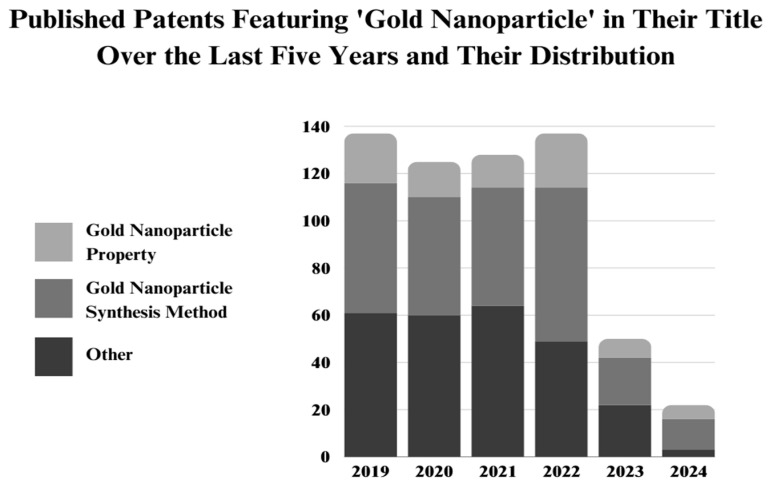
Graph representing the number of patents containing keywords that are related to properties and synthesis methods in comparison to the total number of patents published from 2019 to 2024 [381].

On the other hand, the distribution of patents registered in the last 5 years shows similarity in terms of the involvement of properties and synthesis when compared to the published articles on gold NPs (Figure 10). From 2019 to 2022, there is consistent patent registration, suggesting the involvement of gold NPs in various fields. The fact that properties and synthesis methods in these patents comprise nearly 50% of total patent registrations further highlights the development of gold NPs in these areas. The distribution of patent registrations is consistent with the distribution of published articles in recent years (Figure 1). One noticeable trend is the drop in 2023, with a similar potential for 2024. The same decline is also observed in the number of published papers, which may indicate a shift in research focus or a saturation where current challenges hinder research progress. At this point, the importance of synthesis methods should be highlighted, especially with their constant involvement (nearly 30%) in both patent registrations and published articles. Despite the visible drop, gold NP synthesis methods remain a major research area, indicating their importance in the future of gold NPs.

All in all, the manufacturing, characterization, toxicity, and physical and chemical characteristics of gold NPs are all covered in detail in this article. It discusses how to characterize NPs and the variables that influence their creation. In-depth toxicity analyses, surface functionalization techniques, quantitative phytochemical reduction kinetics, and more profound mechanistic understanding are all provided by recent gold NP research. Gold NPs are typically thought to be non-toxic; however at certain doses, especially in drug delivery and anticancer research, they can have negative consequences. By creating innovative surface modification and functionalization methods, future studies in green synthesis can address any toxicity issues. Thorough research on the best dosages for administration can increase the precision of toxicity evaluations. These methods improve experimental results in addition to addressing toxicity issues with gold NPs. Green nanotechnology will offer a thorough comprehension of these components as well as state-of-the-art technology for efficient use in the biological and pharmaceutical industries. Developing green commercialization methods for safe and sustainable usage requires an understanding of the biological and environmental consequences of gold NPs.

## 6. Conclusions

Gold NPs, a category of metal NPs, possess considerable promise in biological domains owing to their wide range of forms and dimensions, spanning from 1 to 100 nm. Known for their facile synthesis, surface modification, biocompatibility, non-toxicity, high surface-to-volume ratio, and size tunability, gold NPs find extensive use in diverse biological applications. Because of their strong LSPR, gold NPs have revolutionized biosensing, bioimaging, cancer research, and photothermal and photodynamic therapy. They are useful in medicinal applications because of their simple functionalization with a variety of ligands, which enables targeted administration. They are also perfect for creating antioxidants and antibacterials due to their electrical and heat management capabilities.

However, there are certain areas that lack background research in the literature on gold NPs. Gold NP-based tissue engineering is an unnoticed field where enhancing antibacterial activity through surface modification offers a promising approach for developing novel research. Future studies should not only investigate changes in the surface chemistry of gold NPs during modification to observe how antimicrobial activity is affected but also focus on integrating these improvements into regenerative applications. Given their promising properties, gold NP-based tissue engineering studies deserve greater attention. Moreover, their role in the field of neurobiology has also attracted less attention compared to the mentioned areas. The ability of small-sized gold NPs to overcome the BBB, coupled with their superior delivery characteristics, facilitates the efficient release of drugs into targeted areas. Hence, they can be further employed to develop novel solutions in the treatment of neurodegenerative diseases. Since physical and chemical synthesis requires the use of hazardous materials, green synthesis—which uses plants and microbes—is garnering interest in the scientific community. In addition, effective characterization approaches are therefore necessary, since factors impacting NP quality and quantity are critical to their applications in the environmental, electrical, medicinal, and drug delivery sectors.

In this article, we provided a detailed overview of the physical and chemical properties, production, characterization, and toxicity of gold NPs. This review covers the methods for characterizing NPs, as well as the factors that affect their production. More in-depth toxicity studies, distinct surface functionalization methods, quantitative phytochemical reduction kinetics, and deeper mechanistic insights have all been provided by recent research on gold NPs, which has improved our understanding and may offer a more complete picture of their molecular and functional aspects. While gold NPs are generally regarded as non-toxic in many applications, especially in drug delivery and anticancer studies, they can lead to adverse effects at certain concentrations. Therefore, future studies investigating potential toxicity, particularly using green synthesis, can focus on developing novel surface modification and functionalization techniques. In addition, detailed studies that determine optimal administration doses can further improve the accuracy of toxicity assessments. Such approaches not only address concerns related to gold NP toxicity but also hold significant potential for enhancing experimental outcomes. In this manner, future studies in green nanotechnology will offer a thorough comprehension of these elements, as well as cutting-edge technology for effective uses in the pharmaceutical and biological sectors. For the purpose of creating green commercialization strategies for the safe and sustainable use of gold NPs in a variety of industries, it is essential to comprehend the biological and environmental effects of these particles.

Given these factors, it is crucial to evaluate and alleviate any potential adverse effects in order to ensure safer outcomes. All of these characteristics point to the significant role that gold NPs play in nanotechnology, where their special set of qualities not only encourages novel applications but also emphasizes the necessity of more research.

## Data Availability

Not applicable.
